# The Cuprizone Model: Dos and Do Nots

**DOI:** 10.3390/cells9040843

**Published:** 2020-03-31

**Authors:** Jiangshan Zhan, Teresa Mann, Sarah Joost, Newshan Behrangi, Marcus Frank, Markus Kipp

**Affiliations:** 1 Institute of Anatomy, Rostock University Medical Center, Gertrudenstrasse 9, 18057 Rostock, Germany; Jiangshan.Zhan@med.uni-rostock.de (J.Z.); Teresa.Mann@med.uni-rostock.de (T.M.); Sarah.Joost@med.uni-rostock.de (S.J.); Newshan.Behrangi@med.uni-rostock.de (N.B.); 2 Medical Biology and Electron Microscopy Center, Rostock University Medical Center, Strempelstraße 14, 18057 Rostock, Germany

**Keywords:** multiple sclerosis, cuprizone model, histological analyses

## Abstract

Multiple sclerosis (MS) is a chronic inflammatory demyelinating disease of the central nervous system. Various pre-clinical models with different specific features of the disease are available to study MS pathogenesis and to develop new therapeutic options. During the last decade, the model of toxic demyelination induced by cuprizone has become more and more popular, and it has contributed substantially to our understanding of distinct yet important aspects of the MS pathology. Here, we aim to provide a practical guide on how to use the cuprizone model and which pitfalls should be avoided.

## 1. Introduction

Multiple sclerosis (MS) is a frequent disease of the central nervous system (CNS) affecting predominantly young adults. On the histopathological level, the disease is characterized by focal white and grey matter demyelination, caused by the interplay of brain resident cells (such as microglia and astrocytes) and peripheral immune cells (such as lymphocytes and monocytes). Besides demyelination, the focal and diffuse invasion and (re-) activation of these immune cells results in damage to nerve cells [1]. Of note, virtually all neuronal subcellular structures can be destroyed in the brains of MS patients, including axons, dendrites or synaptic spines [2,3,4,5]. Eventually, entire nerve cells can get lost [5,6]. On the clinical level, distinct disease courses can be distinguished: at the beginning of the disease, most patients suffer from the sudden occurrence of new neurological symptoms, which usually disappear after several weeks [7]. This initial disease course is called relapsing remitting MS (RRMS), which means that symptoms appear (i.e., a relapse) and then fade away, either partially or completely (i.e., remitting). By definition, during the RRMS disease phase, the level of clinical disability remains stable in between two relapses. After several years (10–15 years), the frequency of relapses decreases, and the patients clinically deteriorate independent of the relapses. This so-called secondary progressive MS (SPMS) course is thus characterized by chronically progressive clinical worsening over time, with or without superimposed relapses. In about 15% of the patients, the disease is characterized by neurologic worsening (accumulation of disability) from the onset of symptoms, without early relapses or remissions, called primary progressive MS (PPMS).

On the pathological level, the RRMS disease course is characterized by recurrent episodes of inflammatory white matter demyelination. These inflammatory events are probably driven by peripheral, autoreactive immune cells, which invade the CNS parenchyma via the blood–brain or blood–liquor barrier. Consequently, drugs that interfere with leukocyte travelling and invasion, such as Natalizumab or Fingolimod, significantly decrease the frequency of relapses during the RRMS disease phase. 

Although patients can clinically recover completely from a relapse, this does not necessarily mean that there is no residual damage to the brain parenchyma. Such residual damage can be triggered by (i) instant and (ii) delayed neuronal damage. (i) From pre-clinical and post-mortem studies, there is ample evidence that focal inflammatory demyelination induces neurodegeneration, such as axonal transection or neuronal apoptosis [4,5,8]. In the classical autoimmune model of MS, experimental autoimmune encephalomyelitis (EAE), encephalitogenic T helper 17 (Th_17_) cells have been shown to form direct physical contacts with neurons during the development of inflammatory lesions [8]. These Th_17_ cells eventually induce a marked, localized, and partially reversible raise in intra-axonal Ca^2+^ concentrations that might lead to axonal transection or neuronal soma degeneration. Of note, Th_1_ cells were much less efficient at killing neurons through this mechanism, but have been shown to contribute to neuronal damage by releasing tumor necrosis factor-related, apoptosis-inducing ligand, and by activating the pro-apoptotic mediator caspase-3 in neurons [9]. (ii) Besides such immediate harmful events, delayed degenerative processes can be triggered by the focal lesions. For example, if the focally destroyed myelin sheath cannot be repaired (i.e., remyelination) different biochemical mechanisms can trigger delayed axonal degeneration among an increased energy demand from impulse conduction along excitable demyelinated axons [10], lack of axonal trophic support by oligodendrocytes [11], a lethal rise in intra-axonal calcium levels [12], or a higher vulnerability of demyelinated axons against cytotoxic substances. Beyond this, white and grey matter areas distant to the focal lesion side show subtle yet important signs of tissue damage, such as microglia activation, abnormal blood vessels, increased expression of genes related to proteolytic processing, or a decreased expression of genes regulating oligodendrocyte survival [13,14]. Since these white and grey matter regions are not “normal” (i.e., healthy) they are described as normal-appearing white and grey matter (NAWM and NAGM, respectively). To conclude, although neurological function can fully recover after a relapse, some slow-burning degenerative processes are triggered by focal lesions that are not yet apparent during the early disease phase. These slow-burning degenerative processes are believed to be driven by local, innate immune cells. Of note, the results of a recent clinical trial suggests that Siponimod, which modulates sphingosine-1 phosphate receptor activities [15], might ameliorate such delayed neurodegenerative processes by modulating sphingosine-1 phosphate receptor signaling expressed in astrocytes, microglia, and oligodendrocytes [16].

During progression of the disease, two fundamental pathogenetic factors change: first, the activity of the adaptive immune system decreases, which clinically results in a lower frequency of clinically detectable relapses [17]. Why the adaptive (and maybe also the innate) immune system becomes less active during disease progression is currently unknown, but immunosenescence probably plays an important role [18]. Second, the slow-burning degenerative process reaches a certain threshold and becomes clinically apparent. Two mechanisms likely play a role in the delayed clinical manifestation of this slow-burning neurodegeneration. On the one hand, the function of damaged or degenerated neurons can be carried from neighboring neurons, a process called neuronal plasticity [19,20]. On the other hand, the destruction of single or a group of neurons does not necessarily result in overt clinical deficits. This is maybe best illustrated by the fact that as many as 80% of the dopaminergic neurons may be lost before clinical symptoms are apparent in affected Parkinson patients. Another, more plastic example might be helpful: imagine in front of you is a container with 1000 balls. If somebody takes out one single ball it is rather unlikely that this will be recognized. However, if just two balls are left, most people will certainly recognize if another ball is taken out of the container. During the RRMS disease stage, the initial loss of neuronal structures is not recognized by the patient or, in other words, does not lead to overt clinical deficits. Later during the disease, at the transition phase from RRMS to SPMS, when many neurons are already lost, the subsequent damage to additional neuronal structures results in accumulating and overt clinical deficits. 

To summarize this part of the article, adaptive immunity is the driving force during RRMS, whereas brain resident innate immune cells are believed to cause tissue damage during the progressive phase of the disease. Beyond that, the loss of the myelin sheath makes axons more vulnerable, and therefore, failure of remyelination aggravates disease progression. Current strategies to ameliorate or even halt disease progression in SPMS and PPMS patients are (i) the strengthening of neuroprotective pathways, (ii) amelioration of diffuse innate immune responses, and (iii) the induction of remyelination. 

## 2. Characteristics of the Cuprizone Model

In the following chapter we will briefly introduce the cuprizone model, a toxin-induced demyelination model [21,22]. We will then focus on technical aspects of this model, and thus hope to provide the unexperienced scientist guidelines how to work effectively with this pre-clinical MS tool.

Oral intoxication with the copper-chelator cuprizone induces oligodendrocyte apoptosis within a few days, which is closely followed by the activation of the innate immune cells in the brain, i.e., astrocytes and microglia, finally leading to demyelination of distinct white and grey matter brain areas. Although minor damage to the blood–brain barrier has been described in this model [23], cells of the adaptive immune system, particularly T- and B-cells, are believed to play a non-dominant role during cuprizone-induced demyelination [24,25]. This model thus reflects several important characteristics of the progressive MS disease course. With the cuprizone model, two main aspects related to the MS pathology can be investigated: first, mechanisms underlying innate immune cell-driven myelin and axonal degeneration, and second, remyelination of the demyelinated axons. 

To induce acute demyelination, young adult mice are intoxicated with cuprizone per os for 5 to 6 weeks. In our hands, consistent and intense demyelination is obtained after a 5 week intoxication protocol (0.25% cuprizone, mixed into ground rodent chow). If animals are provided normal chow after week 5 (i.e., acute demyelination), spontaneous endogenous remyelination occurs. In case the cuprizone intoxication period is prolonged (i.e., chronic demyelination), this endogenous regenerative process is severely disturbed [26,27,28]. Most labs, including ours, perform a 12–13 week cuprizone intoxication period to obtain chronically demyelinated lesions. Although remyelination occurs after a chronic cuprizone-induced demyelination as well, myelin repair is significantly slower [26,28]. It is important to notice that after acute cuprizone-induced demyelination, one should not investigate the potency of a pharmaceutical compound to induce remyelination, but rather can assess its potency to accelerate an ongoing remyelinating process, or to inhibit it [29,30]. To study remyelination in the non-supportive environment, one can either apply the chronic cuprizone model or, as demonstrated several years ago, one can use aged animals [31], probably because of the induction of senescence-associated inhibitors of oligodendrocyte differentiation [32,33]. 

After having provided a general introduction to the cuprizone model, we now will discuss histopathological characteristics during the different experimental intoxication periods.

### 2.1. Week 1

**Figure 1:**
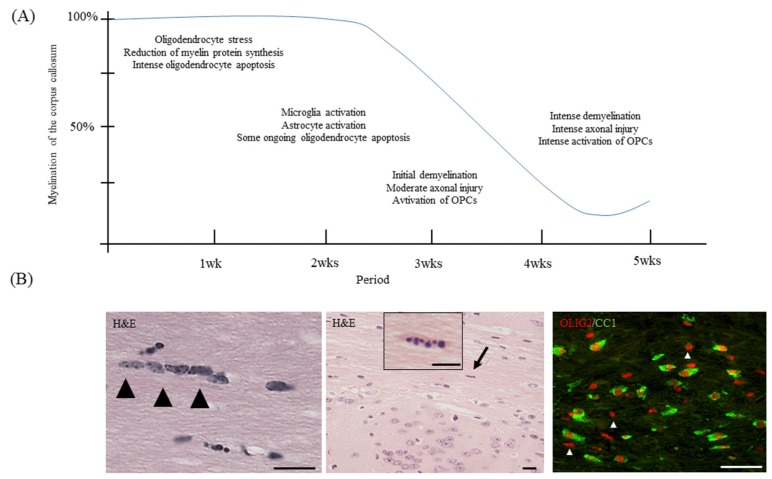
Hallmarks of the cuprizone model. (**A**) Schematic drawing illustrating pathological hallmarks during the course of cuprizone-induced demyelination. The blue line illustrates the levels of myelination. (**B**) The left image illustrates interfascicular oligodendrocytes (arrowheads) in the corpus callosum of a control mouse. The center image illustrates the appearance of an apoptotic cell (arrow) after 1 week of cuprizone intoxication. The right image illustrates mature OLIG2^+^/CC1^+^ oligodendrocytes and pre-mature OLIG2^+^/CC1^−^ oligodendrocytes (white arrowheads). Scale bar = 10 µm. Abbreviations: oligodendrocyte transcription factor 2 (OLIG2), adenomatous polyposis coli gene clone CC1 (CC1).

**Figure 2:**
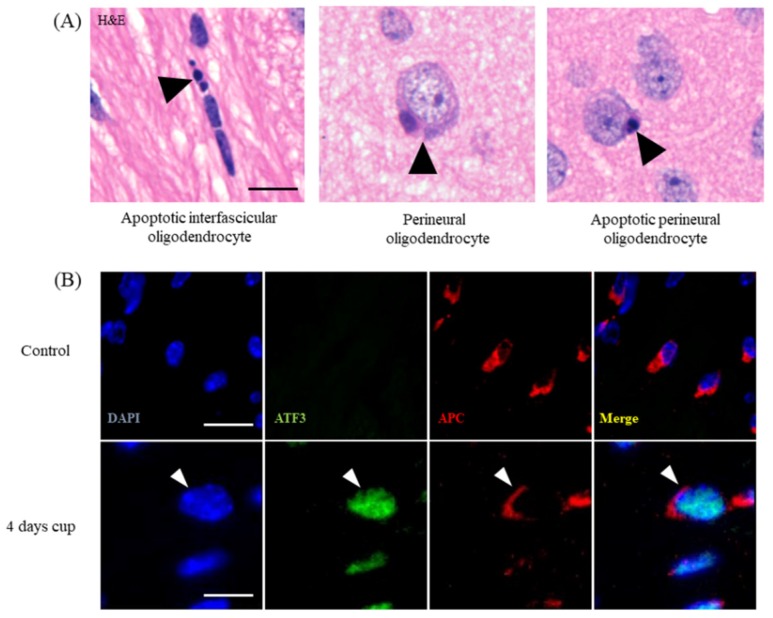
Oligodendrocyte stress and degeneration. (**A**) Different appearances of apoptotic oligodendrocytes in the white matter corpus callosum and grey matter cortex. The left image illustrates the appearance of an apoptotic cell (arrowhead) in the corpus callosum after 1 week of cuprizone intoxication. The center image illustrates a perineuronal oligodendrocyte in the cortex of a control mouse. The right image illustrates an apoptotic perineuronal oligodendrocyte. Perineuronal oligodendrocytes are also called “satellite oligodendrocytes”. Scale bar = 10 µm. (**B**) Expression of the stress transcription factor ATF3 in control animals and in mice intoxicated with cuprizone for 4 days. White arrowheads highlight a stressed APC^+^ oligodendrocyte. Scale bar = 10 µm (upper row) and 5 µm (lower row). Abbreviations: activating transcription factor 3 (ATF3).

**Figure 3:**
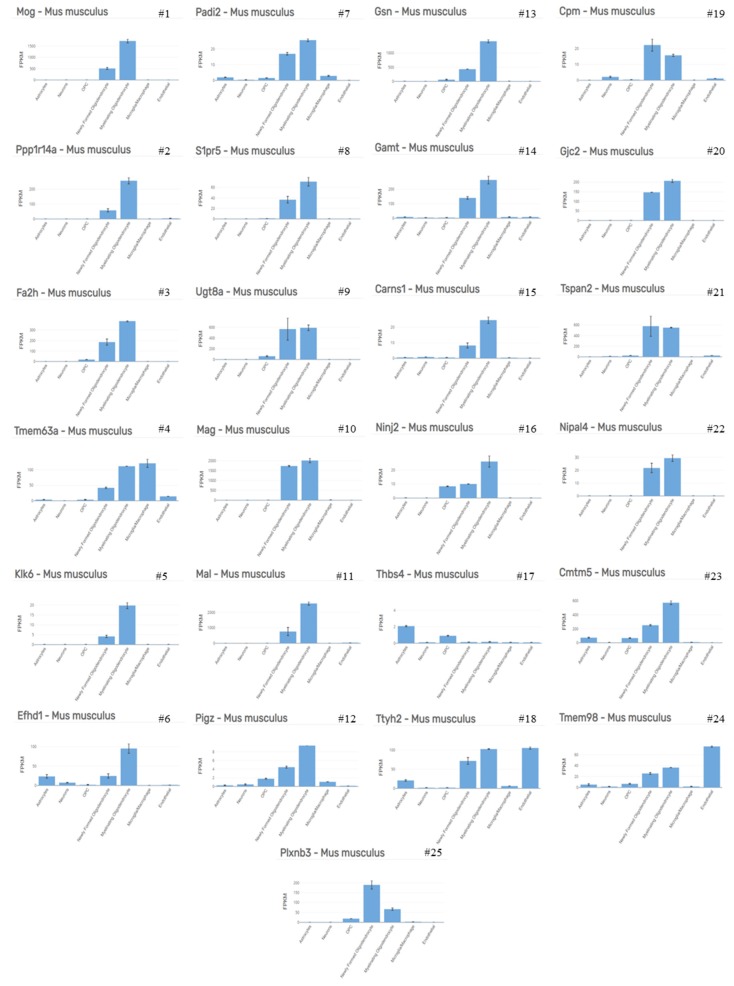
Cellular expression signature. Cellular enrichment of the top 25 downregulated mRNAs as shown in Table 1, retrieved from Brain RNA-Seq database [36]; # represents rank number. Full-size image see Supplementary Figure S1. Abbreviations: myelin oligodendrocyte glycoprotein (*Mog*), protein phosphatase 1 regulatory inhibitor subunit 14A (*Ppp1r14a*), fatty acid 2-hydroxylase (*Fa2h*), transmembrane protein 63A (*Tmem63a*), kallikrein related peptidase 6 (*Klk6*), EF-hand domain family member D1 (*Efhd1*), peptidyl arginine deiminase 2 (*Padi2*), sphingosine-1-phosphate receptor 5 (*S1pr5*), UDP galactosyltransferase 8A (*Ugt8a*), myelin-associated glycoprotein (*Mag*), myelin and lymphocyte protein (*Mal*), phosphatidylinositol glycan anchor biosynthesis class Z (*Pigz*), gelsolin (*Gsn*), guanidinoacetate N-methyltransferase (*Gamt*), carnosine synthase 1 (*Carns1*), nerve injury-induced protein 2 (*Ninj2*), thrombospondin 4 (*Thbs4*), tweety family member 2 (*Ttyh2*), carboxypeptidase M (*Cpm*), gap junction protein gamma 2 (*Gjc2*), tetraspanin 2 (*Tspan2*), NIPA-like domain containing 4 (*Nipal4*), CKLF-like MARVEL transmembrane domain containing 5 (*Cmtm5*), transmembrane protein 98 (*Tmem98*), and plexin B3 (*Plxnb3*).

As demonstrated by several groups, the first apoptotic oligodendrocytes appear days after initiation of the cuprizone intoxication protocol (See Figure 1A for a schematic illustration of the course of cuprizone-induced demyelination and Figure 1B, as well as Figure 2, for the appearance of apoptotic oligodendrocytes). In a recently published work, we compared the mRNA expression levels in the white matter tract corpus callosum isolated from control mice and animals intoxicated with cuprizone for 2 days by gene array analysis [34,35]. As soon as 48 h after initiation of the cuprizone intoxication, we found the expression levels of numerous mRNAs increased or decreased. The most impressive finding was that those mRNA species which were found to be reduced in cuprizone-intoxicated mice are reported to be mostly expressed by oligodendrocytes. As shown in Table 1 and Figure 3, 22 out of the top 25 downregulated mRNAs were found to be enriched in oligodendrocytes (cellular enrichment was retrieved from Brain RNA-Seq database [36]). In contrast, the top 25 upregulated mRNAs were found to be enriched in various cell types, such as astrocytes, microglia/macrophages, and endothelial cells. Interestingly, two out of the top 25 induced genes were found to be enriched in oligodendrocyte progenitor cells (i.e., *Serpina3n* and *Fam46a*), suggesting that oligodendrocyte progenitor cells (OPCs) might also participate in inflammatory responses, as previously suggested [37]. Alternatively, it might be that mature oligodendrocytes re-express proteins expressed during oligodendrocyte development, as suggested for astrocytes [38], or that OPCs are activated early during the course of cuprizone-induced demyelination. Nevertheless, this rough gene array analysis indicates that (i) cuprizone predominantly impairs mature oligodendrocyte homeostasis, and (ii) that other glia cells, such as astrocytes and microglia, but also endothelial cells and OPCs, are activated early in the intoxication period. After a 1 week cuprizone intoxication period, oligodendrocyte loss and microglia/astrocyte activation are clearly evident. The loss of oligodendrocytes can immunohistochemically be investigated by various antibodies. Our lab most commonly uses either anti-oligodendrocyte transcription factor 2 (OLIG2) or anti-adenomatous polyposis coli gene clone CC1 (APC or CC1) antibodies. Both can be reliably used to quantify the loss of mature oligodendrocytes during the early cuprizone intoxication period. However, one must take into consideration two important things: First, anti-OLIG2 antibodies are not specific for mature oligodendrocytes, but are expressed as well in OPCs. However, during early cuprizone-induced intoxication (i.e., after week 1) OPCs are not yet proliferating, and thus, the loss of anti-OLIG2^+^ cells is a good estimate for the extent of cuprizone-induced mature oligodendrocyte damage. Second, there have been some reports suggesting that CC1 is not just expressed by mature oligodendrocytes, but that anti-CC1 antibodies can also label activated astrocytes [39,40]. For the cuprizone model at least, CC1 expression in GFAP^+^ astrocytes has been ruled out by the Stangel’s lab [41], suggesting that CC1 is a suitable marker to stain cells of oligodendroglial origin in this model. As demonstrated in Figure 1B (right image), almost all CC1^+^ cells co-express the oligodendrocyte lineage marker protein OLIG2. Alternatively, anti-OLIG2/CC1 double labelling can be performed to quantify the loss of mature oligodendrocyte cell numbers. In that case, OLIG2^+^/CC1^+^ cells can be considered to be mature oligodendrocytes, whereas OLIG2^+^/CC1^−^ cells represent pre-mature ones (white arrowheads in Figure 1B, right image). 

To visualize apoptotic oligodendrocytes is relatively simple, but due to increasingly strict requirements of reviewers and journals, it is sometimes a challenging task. The cheapest and simplest way to visualize apoptotic cells is the hematoxylin and eosin stain staining (H&E). In coronal sections, oligodendrocytes can be identified by their characteristic positioning within the white matter. They are aligned in rows between the nerve fibers of the white matter, and are therefore called interfascicular oligodendrocytes (Figure 1B and Figure 2A). As shown by Buschmann and colleagues, numerous apoptotic oligodendrocytes (i.e., condensed or fragmented nuclei; Figure 1B, arrow) can be seen already after 2 days of cuprizone intoxication [42]. Although double-labelling experiments with, for example, anti-active caspase3 antibodies and an oligodendrocyte marker protein antibody can principally be performed, the blinded quantification of apoptotic bodies is, in our opinion, sufficient to estimate the extent of cuprizone-induced oligodendrocyte apoptosis. Of note, while the identification of apoptotic oligodendrocytes in the corpus callosum in H&E-stained sections is relatively easy, the often perineuronal positing of cortical oligodendrocytes (Figure 2A) complicates the evaluation. However, we would like to point out that to the best of our knowledge, no other cell type than oligodendrocytes have been reported to degenerate during the early cuprizone intoxication period. Thus, apoptotic cells, howsoever visualized (H&E, anti-active caspase 3, Terminal deoxynucleotidyl transferase dUTP nick end labeling (TUNEL), etc.), should be regarded to represent apoptotic oligodendrocytes, at least during the early cuprizone intoxication period. Recent studies from our lab showed that stressed, pre-apoptotic oligodendrocytes can be visualized by using antibodies directed against certain stress-related transcription factors, such as DNA damage-inducible transcript 3 protein (DDIT3 or CHOP) or activating transcription factor 3 (ATF3; see Figure 2B) [34]. Both stainings, thus, provide an elegant way to label the stressed oligodendrocyte population. 

**Figure 4:**
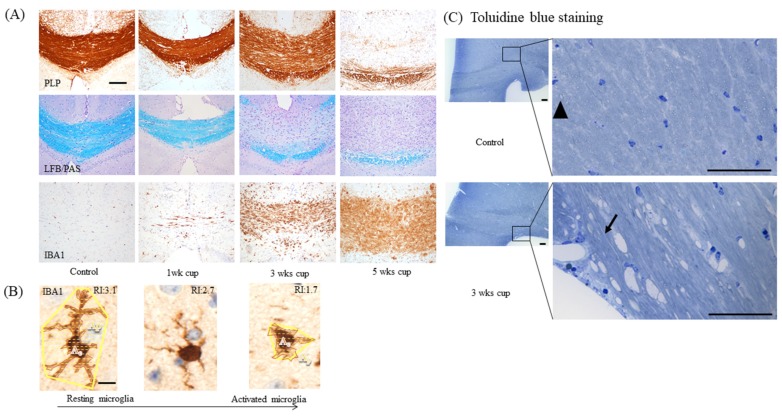
Demyelination and microgliosis. (**A**) Demyelination and microglia activation during the course of cuprizone-induced demyelination. Myelin was visualized by anti-PLP immunohistochemistry (upper row) and LFB/PAS (center row). Microglia activation was visualized by anti-IBA1 immunohistochemistry (lower row). Scale bar = 100 µm. (**B**) The principle of quantifying microglia morphology by calculating a ramification index. The maximum projection area *A_p_* and the cell area *A_c_* are measured. Resting microglia have a relatively large maximum projection area *A_p_*, but a relatively small cell area *A_c_*. In that case, the ramification index *R_i_* has a high value. During their activation, microglia retract their fine processes, and both the cell bodies and processes become hypertrophic. In that case, *R_i_* approaches a value close to 1 (exactly 1 in the case of a perfectly round cell with equal values for *A_p_* and *A_c_*). Scale bar = 5 µm. Abbreviations: myelin proteolipid protein (PLP), Luxol fast blue/periodic acid-Schiff stains (LFB/PAS), and ionized calcium-binding adapter molecule 1 (IBA1). (**C**) Toluidine blue-stained semithin sections in control and cuprizone mice. The arrowhead and arrow indicate transverse or longitudinal sections of an axon, respectively. Scale bar = 50 µm.

To visualize the activation of microglia cells, most laboratories, including ours, use anti-ionized calcium-binding adapter molecule 1 (IBA1) antibodies. At the very beginning, we would like to stress that anti-IBA1 antibodies do not specifically label microglia cells, but rather represent an excellent tool to label monocytes and their derivatives. It is believed that microglia arise from yolk sac erythromyeloid precursors and migrate into the brain parenchyma during early development [43,44,45]. Although microglia represent a heterogeneous cell population [46], they still share several expression profiles with primitive and adult macrophages, including IBA1. Thus, anti-IBA1 antibodies stain microglia cells and invading monocytes. However, in the cuprizone model, and especially during the first week, peripheral monocyte recruitment is negligible, and therefore, numbers of anti-IBA1^+^ cells represent a good estimate for the extent of microglia activation. To quantify microgliosis during this early stage, blinded quantification of cell numbers is a reliable and commonly applied approach. The procedure to do so is relatively simple and straight forward: anti-IBA1 stained sections are digitalized, preferably using a 20-fold or greater objective, the region of interest is outlined (see Section 3.3 in this review article for more comments on the appropriate region of interest in the cuprizone model), and the number of cells with a clearly visible cell body is counted. Finally, the results are given as cells/mm^2^. Of note, a nuclear counterstain, such as haematoxylin for bright-field microscopy or 4′, 6-Diamidin-2-phenylindol (DAPI) for fluorescence-microscopy, should be used to ease the identification of cell bodies. Another frequently applied method is densitometric measurements of anti-IBA1 processed slides. Numerous protocols are available, and different open-source software packages, such as ImageJ, can be used. A less common, but as far as we are concerned, a very powerful and sensitive method to estimate microglia activation during early cuprizone-induced demyelination is the quantification of microglia morphology. As demonstrated in Figure 4B, following this strategy anti-IBA1 stained sections are digitalized, preferably using a 40-fold objective, and the maximum projection area (also called the convex hull; *A_p_* = yellow line in Figure 4B) and the cell area (*A_c_* = brownish area in Figure 4B) are measured and related to each other. This results in the so-called ramification index *R_i_* = *A_p_*/*A_c_*. Resting microglia have a relatively high maximum projection area *A_p_*, but a relatively small cell area *A_c_*. In that case, *R_i_* has a high value. During their activation, microglia retract their fine processes, and both the cell bodies and processes become hypertrophic. Activated microglia thus have a smaller maximum projection area *A_p_* but a bigger cell area *A_c_*. In that case, *R_i_* approaches a value close to 1 (exactly 1 in the case of a perfectly round cell with equal values for *A_p_* and *A_c_*). Such measurements are best performed in the deep layer cortex, since the morphology of cells, including microglia in the white matter tract corpus callosum, is somewhat biased by the axonal bundles oriented in parallel. Other ways to visualize the extent of microglia activation are the staining against commonly accepted microglia activation markers, such as MAC-3, also known as CD107b or lysosomal-associated membrane protein 2 (LAMP-2) [47]. 

**Figure 5:**
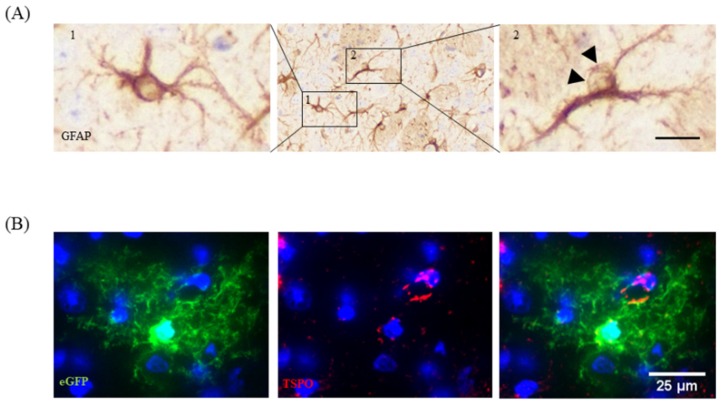
Astrocyte morphology. (**A**) Two anti-GFAP positive cells are demonstrated. Cell 1 shows anti-GFAP immunoreactivity within the entire perinuclear space, which allows for clear definition of the astrocytic cell body. Cell 2 shows anti-GFAP immunoreactivity at just one site of the cell body, making it difficult to clearly delineate the astrocyte cell body (highlighted by arrowheads). Scale bar = 20 µm (center), 10 µm (left, right). (**B**) Expression of the mitochondrial protein TSPO (red) in eGFP-expressing astrocytes. Adopted from [48]. Scale-bar = 25 µm. Abbreviations: glial fibrillary acidic protein (GFAP), translocator protein (TSPO), enhanced green fluorescent protein (eGFP).

To visualize the activation of astrocytes is, unfortunately, more challenging. Most labs use anti-glial fibrillary acidic protein (GFAP) antibodies to label activated astrocytes. Under physiological conditions, the expression levels of GFAP in the murine brain are relatively low, especially in the grey matter cortex region. Although filled with astrocytes, GFAP^+^ cells are hard to delineate in the cortex of healthy mice. If so, GFAP^+^ cells can mainly be found around bigger blood vessels or the superficial pia mater. There, they built up the glia limitans perivascularis and superficialis. Once activated, astrocytes up-regulate the expression of GFAP, and numerous cells become visible. To conclude, anti-GFAP antibodies do not label astrocytes, but rather activated astrocytes. Contrary to anti-IBA1 antibodies, which label the entire cell body and the fine, distal processes of microglia, anti-GFAP antibodies predominantly label the cell body and the thick primary processes of astrocytes. This fact makes morphological measurements somewhat challenging, and we do not recommend this as a standard method of choice to quantify the extent of astrocyte activation. Another circumstance that makes the analysis of astrocyte activation in anti-GFAP-processed brain slides challenging is that within the cell body, GFAP is not evenly distributed, but in many cases spares one site in the cell body (see Figure 5A, image 2). Thus, even in optimally processed sections it is sometimes hard to decide whether or not a GFAP^+^ cell mass indeed represents the astrocytic cell body or simply a thick primary process. One possibility to work around this limitation is the use of transgenic mice, which express a fluorescent protein under the control of an astrocyte-specific promoter. We have recently applied this tool to decide whether or not the translocator protein (TSPO), a protein of the outer mitochondrial membrane, is expressed by astrocytes in the cuprizone model [48]. In this work, human glial fibrillary acidic protein–enhanced green fluorescent protein (hGFAP-eGFP) transgenic mice [49] were used to visualize entire astrocyte cell bodies and processes. To verify TSPO expression in astrocytes, brain slides from cuprizone-treated hGFAP–eGFP-mice were processed for anti-TSPO immunofluorescence staining. As demonstrated in Figure 5B, these mice express eGFP not only within their proximal astrocytic processes, but also within the fine distal processes of astrocytes. Applying this elegant tool, fluorescence labelling clearly showed that the anti-TSPO signal localizes to astrocyte cell bodies. Of note, other marker proteins are known for astrocytes, such as aldehyde dehydrogenase 1 family member L1 (ALDH1L1), vimentin, brain lipid binding protein (BLBP), or the calcium-binding protein S100ß, and can in principal be used to label astrocyte subpopulations. 

### 2.2. Weeks 1–3

As pointed out above, the first week of cuprizone intoxication is dominated by oligodendrocyte apoptosis, paralleled by the early activation of astrocytes and microglia. Anti-myelin stains do not show any abnormalities at this early stage. Between weeks 1 and 3, the degeneration of oligodendrocytes continues, paralleled by a more severe accumulation of astrocytes and microglia cells. At the end of week 3, the first signs of commencing demyelination become evident (see Figure 4A). Of note, the pathology of the myelin sheath is, at week 3, easier to visualize by histochemical stains (e.g., by the Luxol fast blue (LFB)/periodic acid-Schiff (PAS) stain), compared to immunohistochemical approaches. As recently demonstrated by our group, the optical density within the corpus callosum of anti-proteolipid protein (PLP), anti-myelin-associated glycoprotein (MAG), and anti-2′,3′-Cyclic-nucleotide 3′-phosphodiesterase (CNPase) processed sections did show only minor differences between the control and 3 week cuprizone-intoxicated mice [50]. In contrast, myelin pathology was clearly visible by LFB/PAS stains and ultrastructural studies [34]. At this time point, the loss of mature oligodendrocytes is severe and paralleled by first but clear signs of acute axonal pathology.

**Figure 6:**
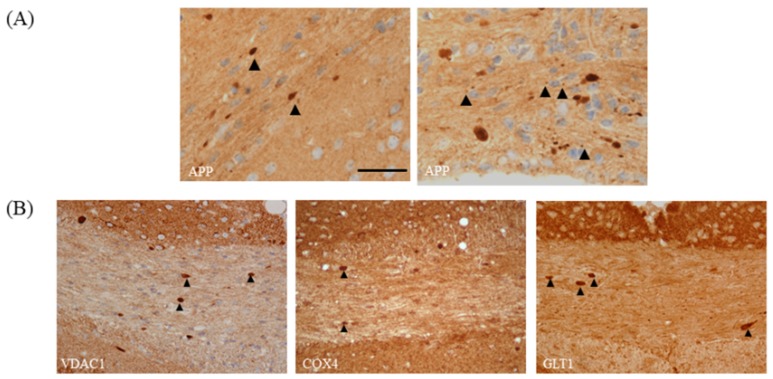
Acute axonal injury. (**A**) Acute axonal injury after 5 weeks of cuprizone intoxication, visualized by anti-APP stains. Arrowheads in the left image highlight APP spheroids. Arrowheads in the right image highlight small APP^+^ particles in close vicinity to a cell nucleus. These small APP^+^ particles might belong to glia cells rather than axons. (**B**) Acute axonal injury visualized by the two mitochondrial-specific antibodies anti-VDAC1 and anti-COX4, as well as the synaptic protein specific antibody anti-GLT1. Arrowheads highlight the sites of acute axonal injury, indicated by a breakdown of the anterograde axonal transport machinery. Scale bar = 30 µm. Abbreviations: amyloid precursor protein (APP), voltage-dependent anion-selective channel 1 (VDAC1), cytochrome c oxidase subunit 4 (COX4), glutamate transporter 1 (GLT1).

As demonstrated in Figure 4A, astrocyte and microglia reactivity are already severe at week 3, which makes their quantification by counting single cells challenging or even impossible. Therefore, we suggest densitometric analyses of staining intensities as the method of choice to quantify astrocyte and microglia activation, at least in the affected corpus callosum. In the cortex and other grey matter regions, microglia and astrocyte activation is less severe [51,52], and therefore the quantification of cell numbers and cell morphology might still be feasible. Acute axonal injury is commonly visualized in anti-amyloid precursor protein (APP)-processed sections. APP is an integral glycoprotein type 1, which is synthetized in the neuronal soma and then transported to the axonal terminal via the anterograde axonal transport machinery [53]. In case of a disturbed axonal transport machinery, APP accumulates at the sites of axonal injury, and can be visualized by immunohistochemistry as spheroids [54,55]. Of note, the focal accumulation of APP has also been observed in MS lesions and other animal models of MS [4,56]. A recent study of our group showed that both vesicular and mitochondrial proteins accumulate as spheroids at sites of acute axonal injury in the cuprizone model [57]. Thus, the visualization of acute axonal injury can as well be performed with antibodies specific for synaptic vesicles (e.g., anti-VGLUT1) or integral mitochondrial proteins (e.g., anti-VDAC1 or anti-COX4). If one quantifies the number of axonal spheroids, a nuclear stain is absolutely required. Both APP and mitochondrial proteins are not just expressed in the axonal compartment, but also in astrocytes, microglia, and oligodendrocytes. To be able to decide whether a “spheroid” belongs to an axon or a glia cell body, the spatial relation to a nucleus is extremely helpful. Just spheroids with no spatial relation to a nucleus should be counted (see Figure 6A for an example). Furthermore, very small dots should also be excluded from the analyses, since these could represent mitochondria or APP in thick glia cell processes.

### 2.3. Weeks 3–5

Between week 3 and week 5, microglia gain their full activation status and phagocytose the myelin sheaths. Astrocytosis, microgliosis, and acute axonal injury become more severe, and demyelination becomes clearly visible by immunohistochemistry. Demyelination can also be demonstrated in semi-thin processed sections (see Figure 4C) or by ultrastructural analyses [58,59,60,61,62]. This demyelination, together with astrocytosis and microgliosis, triggers the activation and recruitment of OPCs. Therefore, it is not surprising that the number of OLIG2^+^ cells after a 5 week cuprizone intoxication period is similar or even increased compared to control animals. At least during the acute stage of cuprizone-induced demyelination, these new OPCs are believed to originate from both the subventricular zones and the corpus callosum parenchyma [63]. While discussing mechanisms involved in oligodendrocyte differentiation, particularly in the cuprizone model, is out of the scope of this article, we refer to previously published articles addressing this important aspect [47,64]. 

Although not in the focus of this review article, numerous studies have demonstrated functional deficits induced by cuprizone intoxication. For example, demyelination of the corpus callosum has been linked with impaired motor coordination [65,66,67,68]. Motor deficits persist or are only partially recovered during remyelination, which is measured by different testing paradigms, such as the motor skill sequence test (MOSS), the rotarod test, or the beam cross test [69,70,71]. Mice also showed impaired spatial memory and a changed social behavior after cuprizone intoxication [65,72,73]. For a more in-depth discussion on behavioral deficits in the cuprizone model, we refer to an excellent recently published article [22].

We finally would like to point out that the perfect animal model for MS does not exist. Due to the manifold and heterogeneous pathological processes in MS, each singular animal model allows us to study very distinct aspects of the disease, rather than its entire complexity. Some of the aspects of progressive MS that can be re-capitulated in the cuprizone model are listed in Table 2. A comparison of histopathological characteristics between different MS animal models is given in [6,74,75].

## 3. Dos and Do Nots in the Cuprizone Model

After having addressed the histological characteristics of the cuprizone model and having discussed how different cellular parameters are best quantified, we next aim to list some “dos and do nots” during experimental planning, conduction, and evaluation.

### 3.1. Animal Weight

In most studies, cuprizone intoxication is realized via per os administration, by mixing the pulverized cuprizone into ground rodent chow. Some labs have also reported intoxicating their mice by oral gavage of solubilized cuprizone. While sex, genetic background, and age of the animals have been identified as important variables for the reproducibility and the extent of cuprizone-induced pathological changes [99,100,101], “weight” as a critical variable for reliable and consistent demyelination has just recently been systematically addressed. In a recent work, we have investigated this issue, and were able to show that animal weight is an important variable for reliable cuprizone-induced demyelination [102]. In our group, we obtain the most reliable results if we order male mice from the vendor at an age of 6–7 weeks with weights ranging from 18 to 20 g. Of note, the weight of the mice should be determined after 1 week of rest, because mice show considerable weight loss due to transport-induced stress. 

Especially in animal facilities with limited space, it is usually not feasible to design the experiment in a way that all mice have a similar weight at the beginning of the cuprizone-intoxication period. Therefore, one should try to balance the weight between the different study groups in such a way that lightweight and heavyweight mice are equally distributed among the groups. The same applies for the sex of the animals. 

### 3.2. Cuprizone Formulation

In order to ensure reproducibility, a standardized cuprizone intoxication protocol is absolutely mandatory. In our lab, cuprizone-containing chow is prepared freshly every day by physically mixing cuprizone into ground rodent chow. Alternative methods, such as the provision of cuprizone-containing pellets [103,104,105], mixing of cuprizone into the drinking water [106], or oral gavage of dissolved cuprizone (personal communication) have successfully been applied. However, the effectiveness and reproducibility of these methods have been unknown until recently. In a recent study, we were interested in whether cuprizone-induced demyelination can be achieved in a reliable and reproducible manner by providing animals with cuprizone-containing pellets rather than preparing cuprizone daily in ground rodent chow. We were clearly able to demonstrate that although the preparation of cuprizone in ground rodent chow is laborious and bears the risk of cuprizone inhalation, it is the method of choice to achieve reproducible, demyelinated white matter lesions [107]. This observation is well in line with a report from Hagemeyer and colleagues. The authors noted that cuprizone-containing pellets, instead of cuprizone in ground chow, failed to induce consistent demyelination [68]. Why cuprizone provided in pellet formulation is not as effective as the ground rodent chow formulation remains to be clarified. It was assumed that cuprizone is heat-sensitive [47], and therefore could be partially deactivated during the pellet pressing procedure. However, Heckers and colleagues recently showed that thermal pretreatment of cuprizone neither abolished its demyelinating effects nor the glial responses in cuprizone-intoxicated mice. Thus, heat exposure does not inactivate cuprizone [108]. We have another theory about why cuprizone is less effective in pellet formulation. It is well known that cuprizone chelates copper [106]. Since copper is present in the pellet, prolonged interaction of cuprizone with this copper might inactivate cuprizone over time. In this context, it is important to notice that in our lab, the cuprizone powder is prepared freshly every day at a concentration of 0.25%. A copper-mediated inactivation would, thus, also be possible during ground rodent chow formulation, in case the mixture is not prepared freshly every day. Although we do not know the exact underlying mechanisms of cuprizone activity loss in pellet formulation, we strongly suggest mixing cuprizone into ground rodent chow and preparing this mixture freshly every day. This is indeed time-consuming, but assures reproducible results and successful experiments. 

### 3.3. Selection of the Region of Interest for Histological Analyses

**Figure 7:**
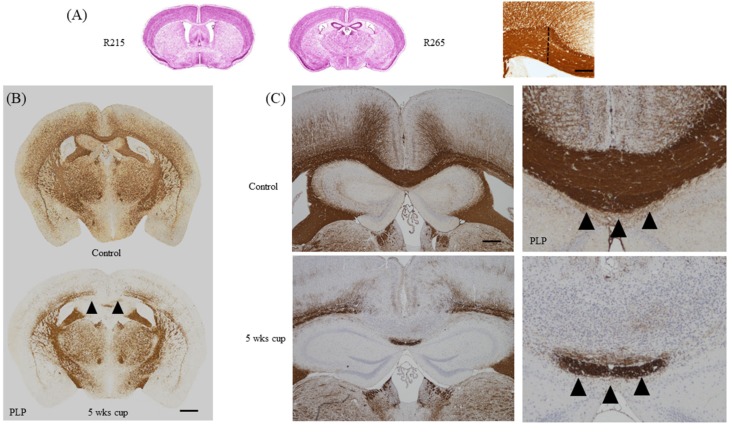
Topographical aspects. (**A**) Principal regions suggested by the authors for histopathological analyses. As shown in the right image, the tip of the cingulum provides a good anatomical border to delineate the lateral border of the midline of the corpus callosum at the level of the anterior commissure (i.e., R215). Scale-bar = 1300 µm (left, center); 150 µm (right). (**B**) Overview of anti-PLP stained sections at the level of the rostral hippocampus from control and 5 week cuprizone-intoxicated mice. The arrowheads highlight severe demyelination of the midline corpus callosum. Scale bar = 650 µm. (**C**) Overview and high magnification of anti-PLP stained sections from control and 5 week cuprizone-intoxicated mice. Arrowheads highlight the fornix, which is somewhat resistant to the cuprizone intoxication. Scale bar = 300 µm (left column); 600 µm (right column). Abbreviations: myelin proteolipid protein (PLP).

Spatio-temporal information about lesion development and progression is an indispensable prerequisite for straightforward de- and remyelination studies. In contrast to the autoimmune-driven EAE model, the site of lesion development in the cuprizone model is highly predictive. However, not all brain regions are equally affected by the toxin. While demyelination is pronounced in the corpus callosum and somato-sensory cortex region [109], other CNS parts, such as the spinal cord [110], the cerebellum [111,112], or the internal capsule [78] are less severely affected. In most studies, the corpus callosum is defined as the region of interest (ROI) in this model. Of note, and this aspect of the model is very important for reliable histological evaluations, not the entire corpus callosum gets demyelinated during the course of the cuprizone intoxication. For example, at the level of the rostral corpus callosum, demyelination is severe and almost complete within the lateral parts, whereas demyelination is incomplete and inconsistent within the midline of the corpus callosum. In contrast, at more occipital levels, such as the body part of the corpus callosum, lateral parts are less severely affected, but demyelination is severe and reproducible within its midline (see Figure 7B, arrowheads). It is therefore mandatory to compare equal brain levels between the different experimental animals. In our lab, we usually analyze two distinct brain regions, which can be outlined very clearly in the coronal processed brain. The first region is at the level of the anterior commissure (slide 53 in the reference Allen Brain atlas [113] or slide 215 in the High Resolution Mouse Brain Atlas by Sidmann et al., [114]). At this brain level, the olfactory limbs of the anterior commissure merge within the midline of the brain, and thus, topographically define a well-demarcated level of the mouse brain (Figure 7A). At the level of the anterior commissure, we suggest separately analyzing both medial and lateral aspects of the corpus callosum. The borders of the cingulum provide a good separation of both parts (dashed line in Figure 7A). The second region is at the level of the rostral hippocampus (slide 64 in the reference Allen Brain atlas or slide 265 in the High Resolution Mouse Brain Atlas by Sidmann et al.), just where the pyramidal layer of the hippocampal cornu ammonis region becomes visible. At the level of the rostral hippocampus, we recommend analyzing the midline of the corpus callosum. In a recently published manuscript from our group, we have tried to mathematically define which part of the midline of the corpus callosum is most severely affected. This study revealed that the first five sectors, compromising a distance of 500 µm from the midline of the corpus callosum, showed most severe demyelination in three different immunohistochemical stains, whereas more laterally-orientated sectors showed incomplete demyelination [50]. The neuroanatomical topography of the corpus callosum and neighboring structures are also highly relevant for an accurate histological evaluation of brain sections. Directly beneath the corpus callosum runs the hippocampal fornix. While demyelination is severe within the medial parts of the corpus callosum, loss of myelin staining intensity is by far less severe in the underlying white matter tract fornix [80]. This study clearly demonstrates that neighboring white matter tracts of the corpus callosum display distinct vulnerability to cuprizone-induced demyelination, and this has direct relevance for evaluation strategies in this frequently used MS animal model.

## 4. Conclusions

The cuprizone model is an elegant and straightforward-to-apply tool to study different aspects of the MS pathology. In particular, different aspects of progressive MS pathological characteristics are nicely recapitulated by the cuprizone-induced pathology. Similarities between the cuprizone model and progressive MS pathology are innately driven myelin and axonal injury, functional activation of oxidative stress pathways [96], and relative preservation of the blood–brain barrier. Although out of the scope of this review article, recent results have shown that the cuprizone model, if combined with active or passive EAE induction, is an elegant tool to study the relevance of brain-intrinsic degenerative events for peripheral immune cell recruitment [115,116]. A better understanding of factors regulating the various histopathological aspects of the cuprizone intoxication will thus potentially pave the way for the development of novel therapeutic strategies in MS patients.

## Figures and Tables

**Table 1:** Top 50 up- and down-regulated genes in 2 day cuprizone vs. control. Top 50 up-regulated (left column) and down-regulated (right column) genes in control versus 2 day cuprizone-intoxicated mice. Data are adopted from [35]. Note that most of the down-regulated genes are predominantly expressed by oligodendrocytes. We identified cell-specific gene expression using the online Brain RNA-seq database [36]. OPC: oligodendrocyte progenitor cell.

Top 50 Up-Regulated Genes	Highest Expressed Cell Type	Top 50 Down-Regulated Genes	Highest Expressed Cell Type
*Atf3*	Microglia/Macrophage	*Mog*	Myelinating Oligodendrocyte
*Tgm1*	Microglia/Macrophage	*Ppp1r14a*	Myelinating Oligodendrocyte
*Cxcl10*	Microglia/Macrophage	*Fa2h*	Myelinating Oligodendrocyte
*Ccl3*	Microglia/Macrophage	*Tmem63a*	Microglia/Macrophage
*Osmr*	Endothelial	*Klk6*	Myelinating Oligodendrocyte
*Adamts1*	Endothelial	*Efhd1*	Myelinating Oligodendrocyte
*Hmox1*	Microglia/Macrophage	*Padi2*	Myelinating Oligodendrocyte
*Plscr2*	Astrocytes	*S1pr5*	Myelinating Oligodendrocyte
*Tnc*	Astrocytes	*Ugt8a*	Myelinating oligodendrocyte
*Cdkn1a*	Endothelial	*Mag*	Myelinating oligodendrocyte
*Serpina3n*	OPC	*Mal*	Myelinating oligodendrocyte
*Cd44*	Astrocytes	*Pigz*	Myelinating oligodendrocyte
*Ddit3*	Microglia/Macrophage	*Gsn*	Myelinating oligodendrocyte
*Serpinb1a*	Myelinating oligodendrocyte	*Gamt*	Myelinating oligodendrocyte
*Cyr61*	Astrocytes	*Carns1*	Myelinating oligodendrocyte
*Myc*	Microglia/Macrophage	*Ninj2*	Myelinating oligodendrocyte
*Tnfrsf12a*	Microglia/Macrophage	*Thbs4*	Astrocytes
*Fam46a*	OPC	*Ttyh2*	Endothelial
*Slc14a1*	Astrocytes	*Cpm*	Newly formed oligodendrocyte
*Fosl1*	Endothelial	*Gjc2*	Myelinating oligodendrocyte
*Dusp10*	Astrocytes	*Tspan2*	Newly formed oligodendrocyte
*Gadd45b*	Microglia/Macrophage	*Nipal4*	Myelinating oligodendrocyte
*Clcf1*	Microglia/Macrophage	*Cmtm5*	Myelinating oligodendrocyte
*Gpr84*	Microglia/Macrophage	*Tmem98*	Endothelial
*Ccl2*	Microglia/Macrophage	*Plxnb3*	Newly Formed Oligodendrocyte
*A2m*	Astrocytes	*Pstpip2*	OPC
*C3ar1*	Microglia/Macrophage	*Slc15a2*	Astrocytes
*Nupr1*	Microglia/Macrophage	*Apln*	Endothelial
*Fos*	Astrocytes	*Ptgds*	Newly formed oligodendrocyte
*1200009O22Rik*	Unknown	*Adssl1*	Myelinating oligodendrocyte
*Ifrd1*	Microglia/Macrophage	*Gstm7*	Endothelial
*Gadd45g*	Astrocytes	*Apod*	Myelinating oligodendrocyte
*Arap2*	Astrocytes	*Lrrn1*	Opc
*Tgif1*	Microglia/Macrophage	*Pllp*	Newly formed oligodendrocyte
*Ifit1*	Endothelial	*Cntn2*	Myelinating oligodendrocyte
*Lilrb4*	Microglia/Macrophage	*Fah*	Myelinating oligodendrocyte
*Hbegf*	Microglia/Macrophage	*Serpind1*	Newly formed oligodendrocyte
*Lcn2*	Microglia/Macrophage	*Agt*	Astrocytes
*Ifit3*	Astrocytes	*Anln*	Myelinating oligodendrocyte
*Myd116*	Microglia/Macrophage	*Cryab*	Myelinating oligodendrocyte
*Stk40*	Microglia/Macrophage	*Mboat1*	Myelinating oligodendrocyte
*Trib3*	Endothelial	*Kndc1*	Newly formed oligodendrocyte
*Gbp2*	Endothelial	*Lrp4*	Astrocytes
*Myd88*	Microglia/Macrophage	*Slc13a3*	Astrocytes
*Tagln2*	Endothelial	*Nmral1*	Myelinating oligodendrocyte
*1810010H24Rik*	OPC	*Fzd2*	Astrocytes
*Slc1a5*	Endothelial	*Paqr6*	Astrocytes
*Phlda1*	OPC	*Gja1*	Astrocytes
*Egr2*	Microglia/Macrophage	*Scd1*	Myelinating oligodendrocyte
*Slc7a11*	Astrocytes	*Fam57a*	Myelinating oligodendrocyte

**Table 2:** Comparison between histopathological hallmarks of progressive MS and the cuprizone model. BBB: blood brain barrier.

Histopathology of Progressive MS	Reference	Cuprizone Model	Reference
Gray matter demyelination	[5,76]	Demyelination in cortical and subcortical structures	[77,78]
Diffuse white matter damage	[76,79]	Demyelination of white matter tracts, especially in the corpus callosum	[78,80]
Axonal damage	[81,82,83]	Axonal damage in demyelinated areas	[56,84]
Minor immune cell infiltration	[85,86]	Little or no infiltration of lymphocytes	[87,88]
Minor BBB integrity loss	[89,90,91]	Minor BBB integrity loss	[22,23]
Profound oxidative injury	[92,93,94]	Accumulation of oxidative damage	[95,96]
Progressive worsening of function	[97,98]	Impaired motor coordination, spatial memory and social behavior	[22,50,65,66,67,68,69,70,72,73]

## Supplementary Materials

The following are available online at https://www.mdpi.com/2073-4409/9/4/843/s1.
