# Supplementary material for: The Cuprizone Model: Dos and Do Nots
Source: Cells. 2020 Mar 31;9(4):843. doi: 10.3390/cells9040843 (PMC7226799; doi:10.3390/cells9040843)

Figure 3

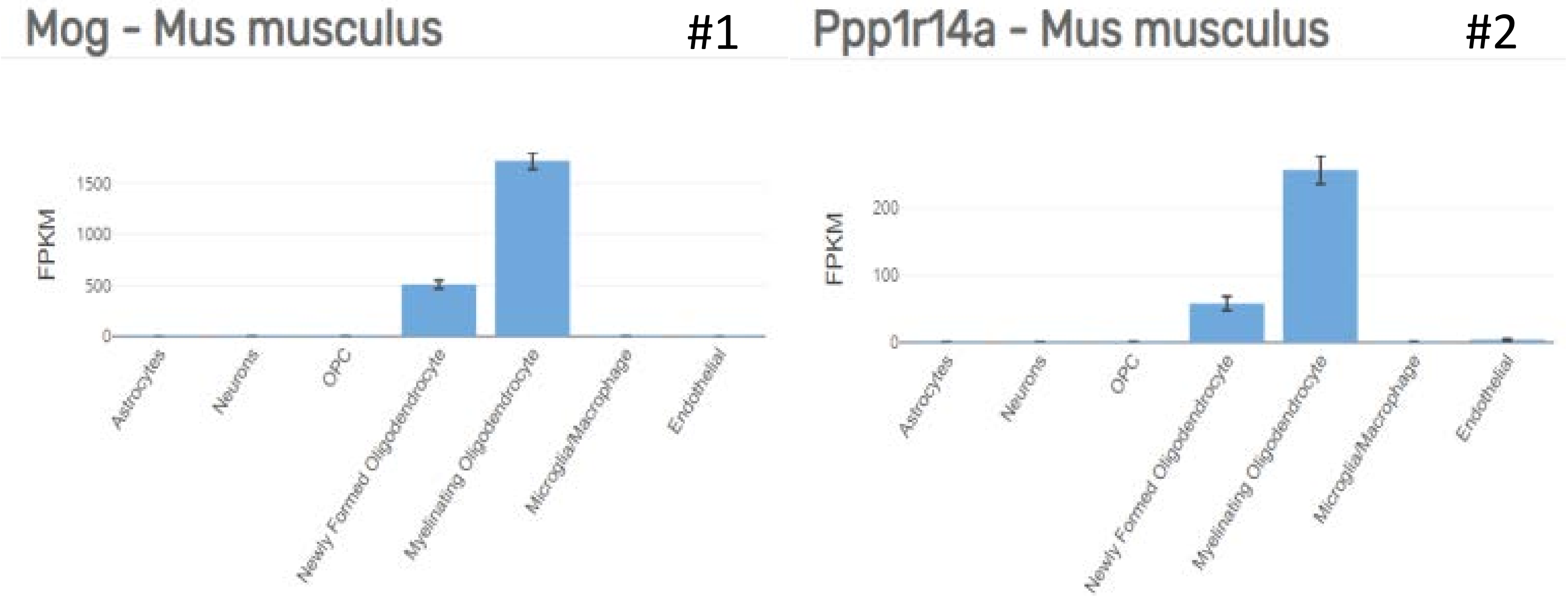

Mog - Mus musculus
#1
FPKM
1500
1000
500
0
Astrocytes
Neurons
OPC
Newly Formed Oligodendrocyte
Myelinating Oligodendrocyte
Microglia/Macrophage
Endothelial
Ppp1r14a - Mus musculus
#2
FPKM
200
100
0
Astrocytes
Neurons
OPC
Newly Formed Oligodendrocyte
Myelinating Oligodendrocyte
Microglia/Macrophage
Endothelial

Fa2h - Mus musculus #3

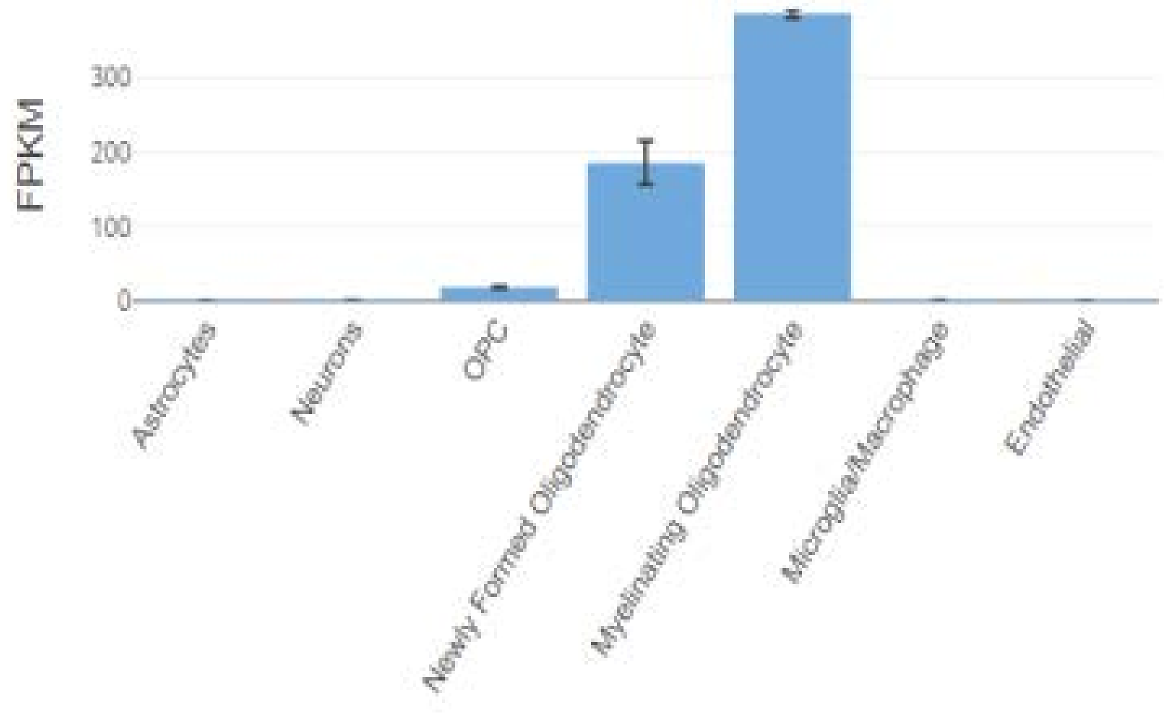

Tmem63a - Mus musculus #4

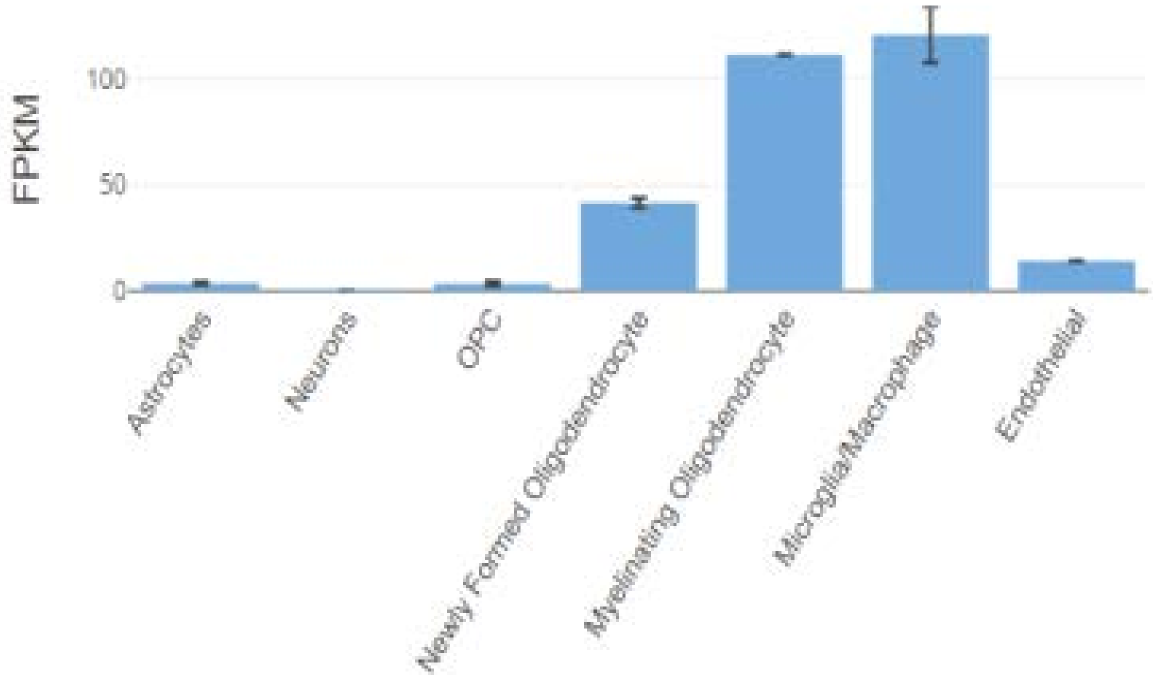

Klk6 - Mus musculus #5

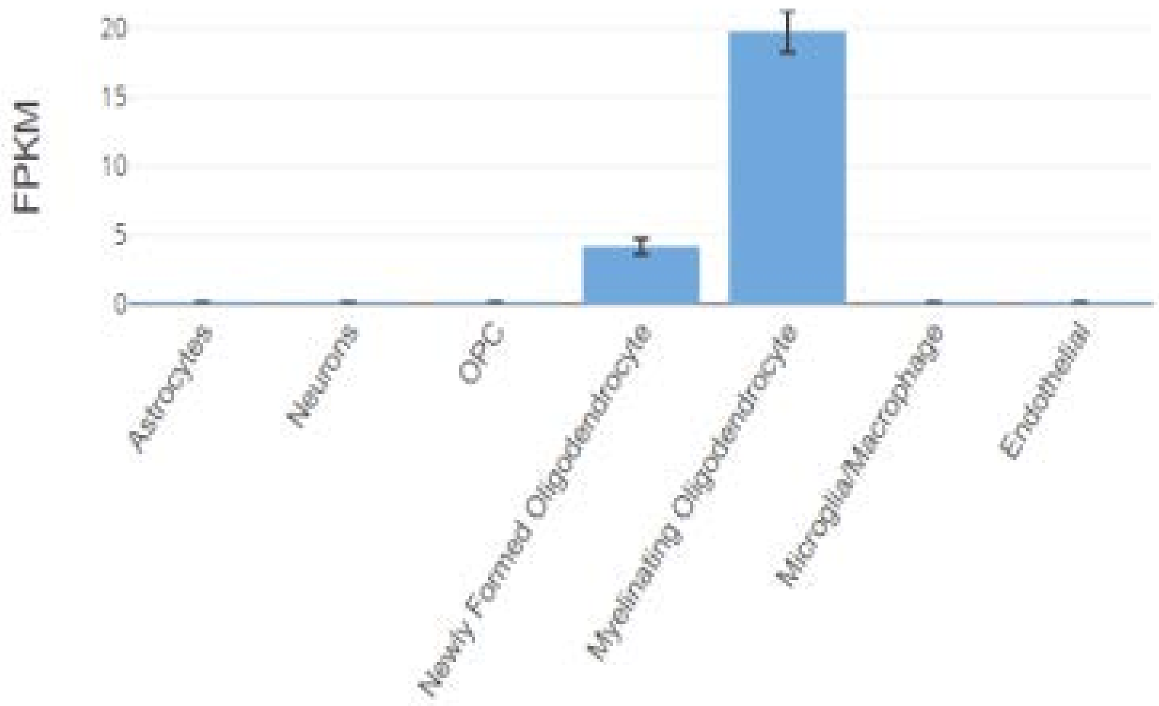

Efhd1 - Mus musculus #6

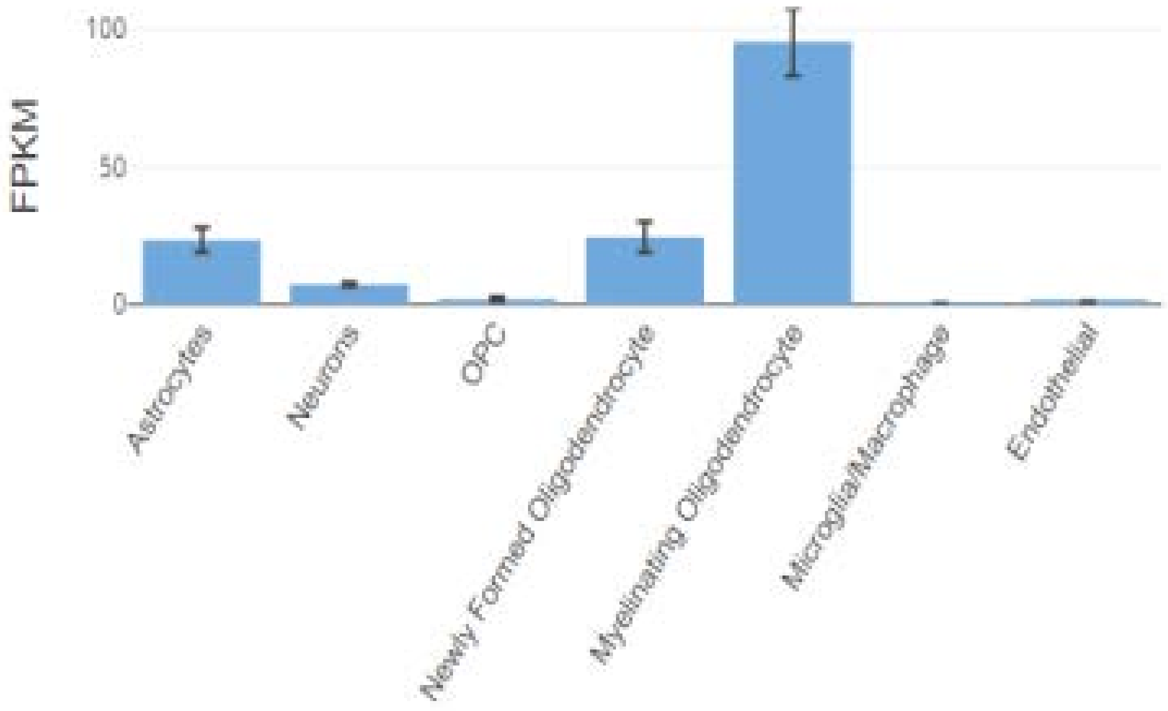

Padi2 - Mus musculus #7

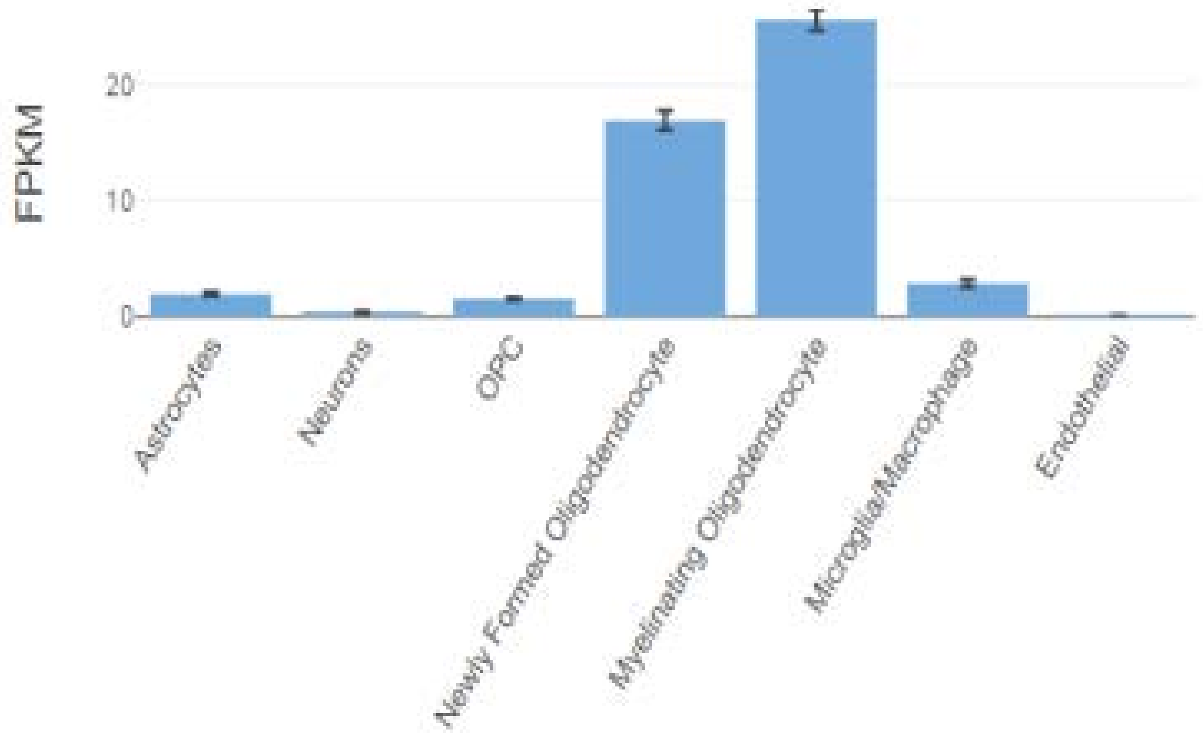

S1pr5 - Mus musculus #8

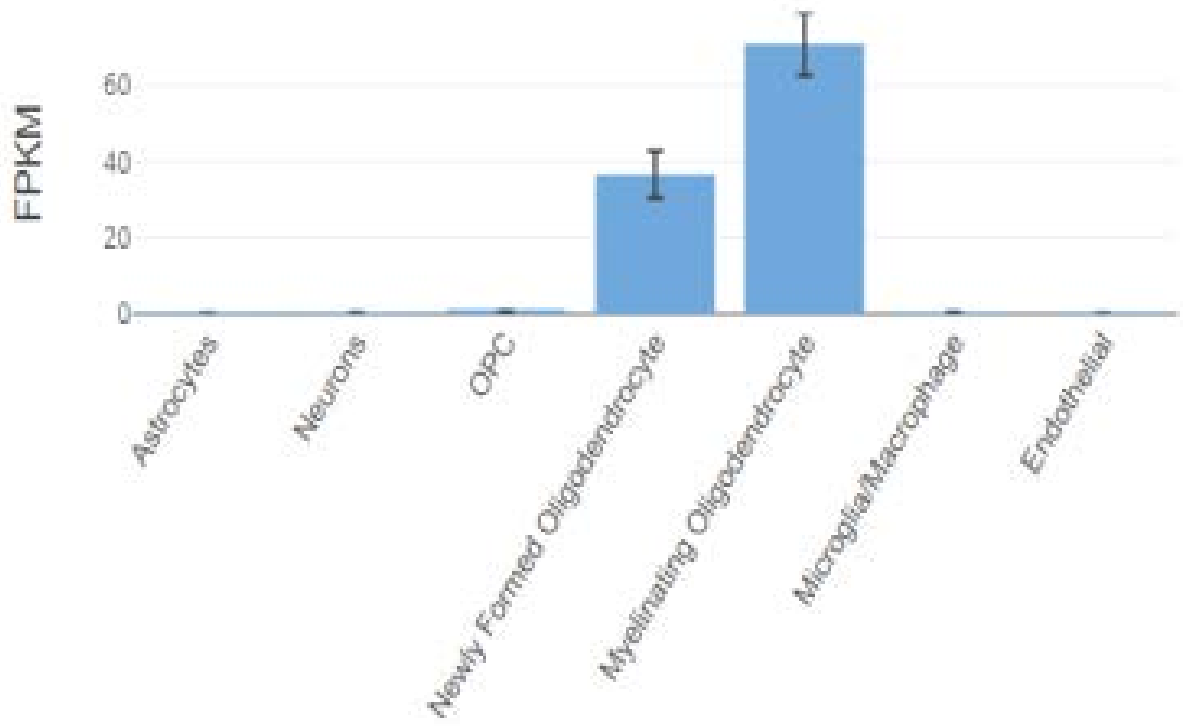

#9

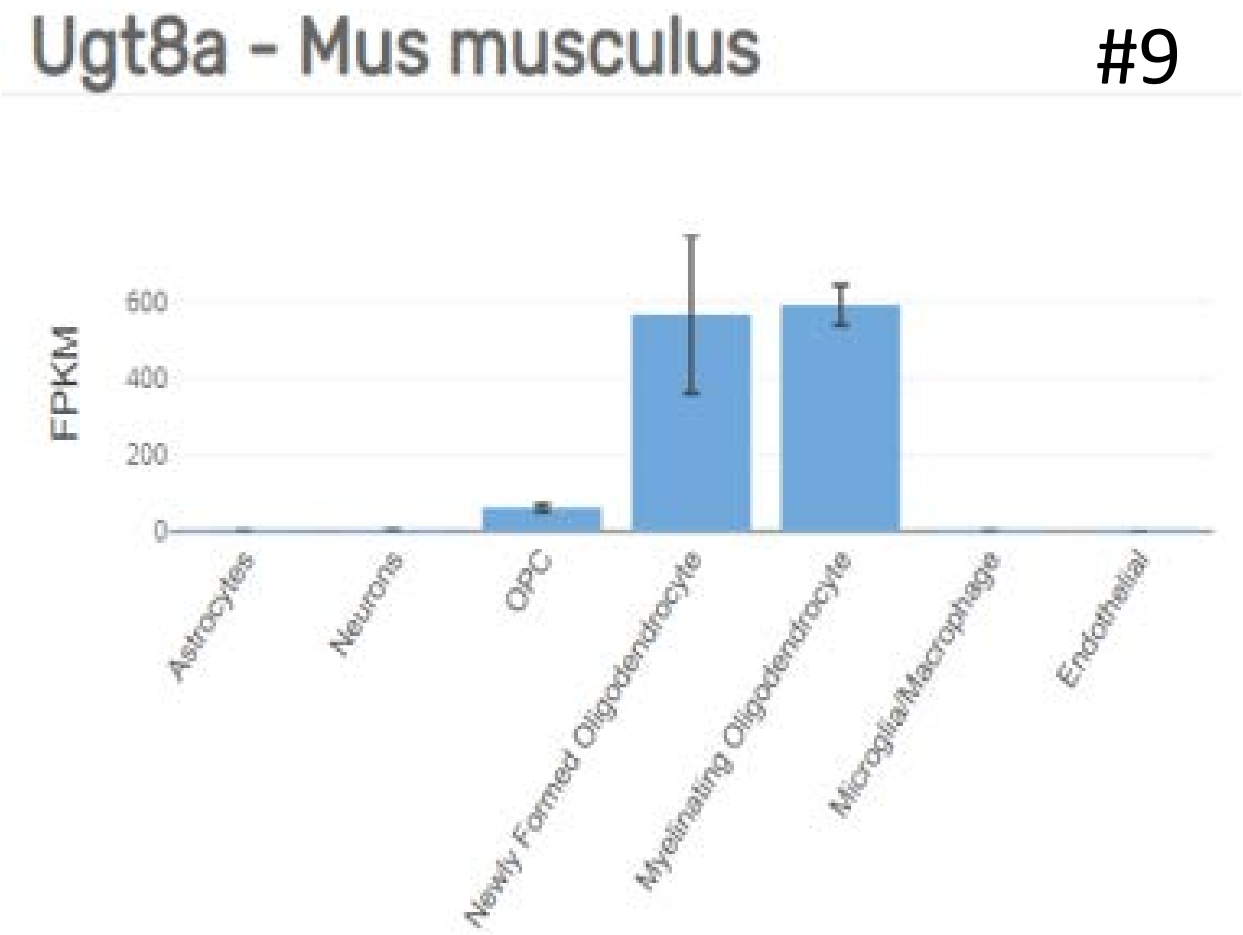
Ugt8a - Mus musculus
FPKM
600
400
200
0
Astrocytes
Neurons
OPC
Newly Formed Oligodendrocyte
Myelinating Oligodendrocyte
Microglia/Macrophage
Endothelial

#10

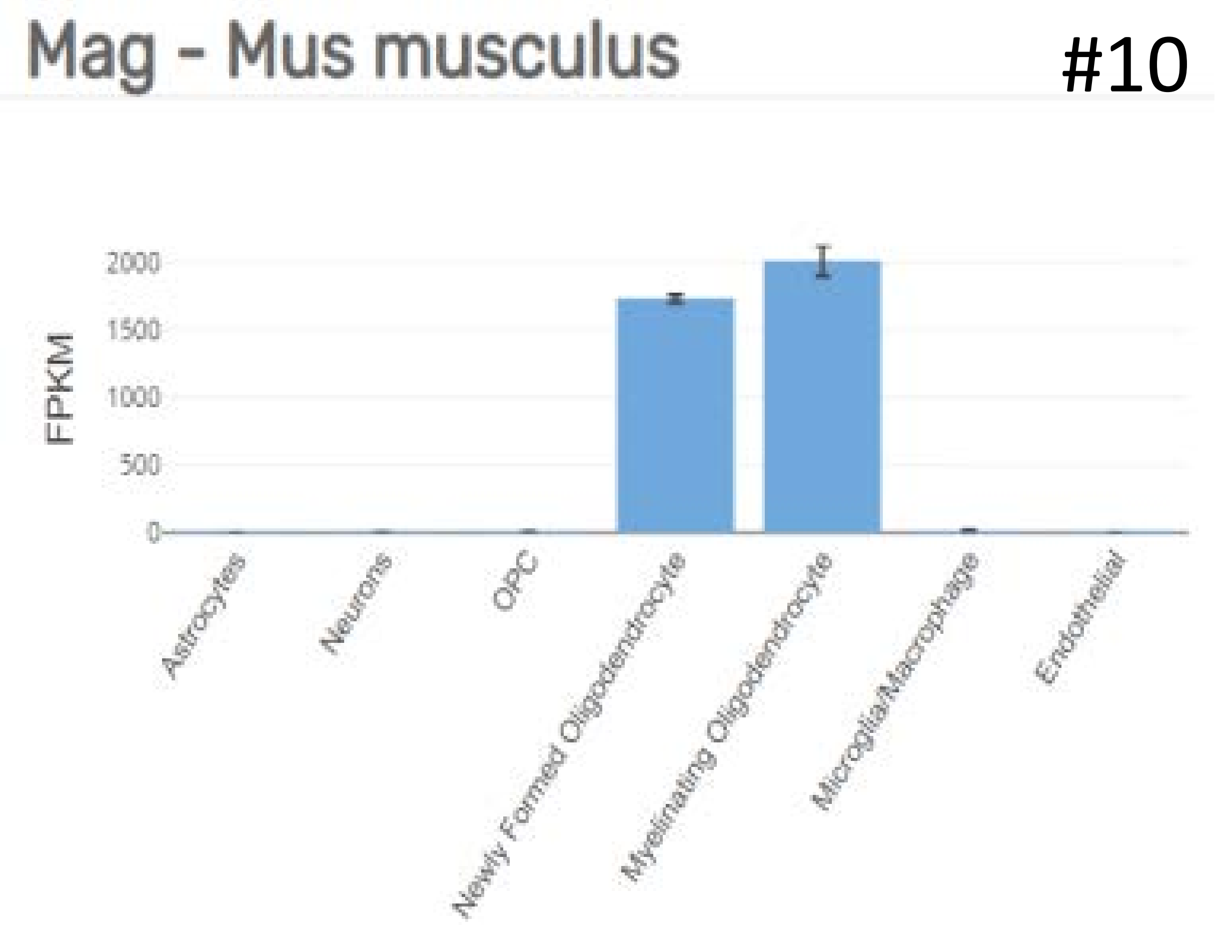
Mag - Mus musculus
FPKM
2000
1500
1000
500
0
Astrocytes
Neurons
OPC
Newly Formed Oligodendrocyte
Myelinating Oligodendrocyte
Microglia/Macrophage
Endothelial

Mal - Mus musculus #11

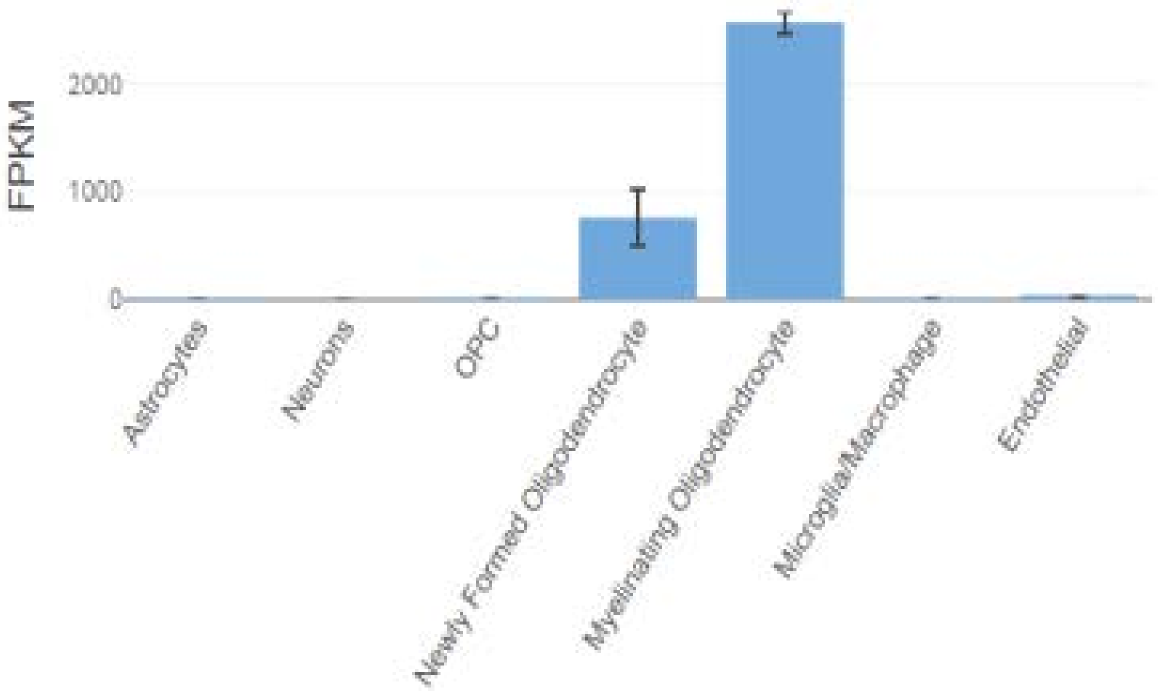

Pigz - Mus musculus #12

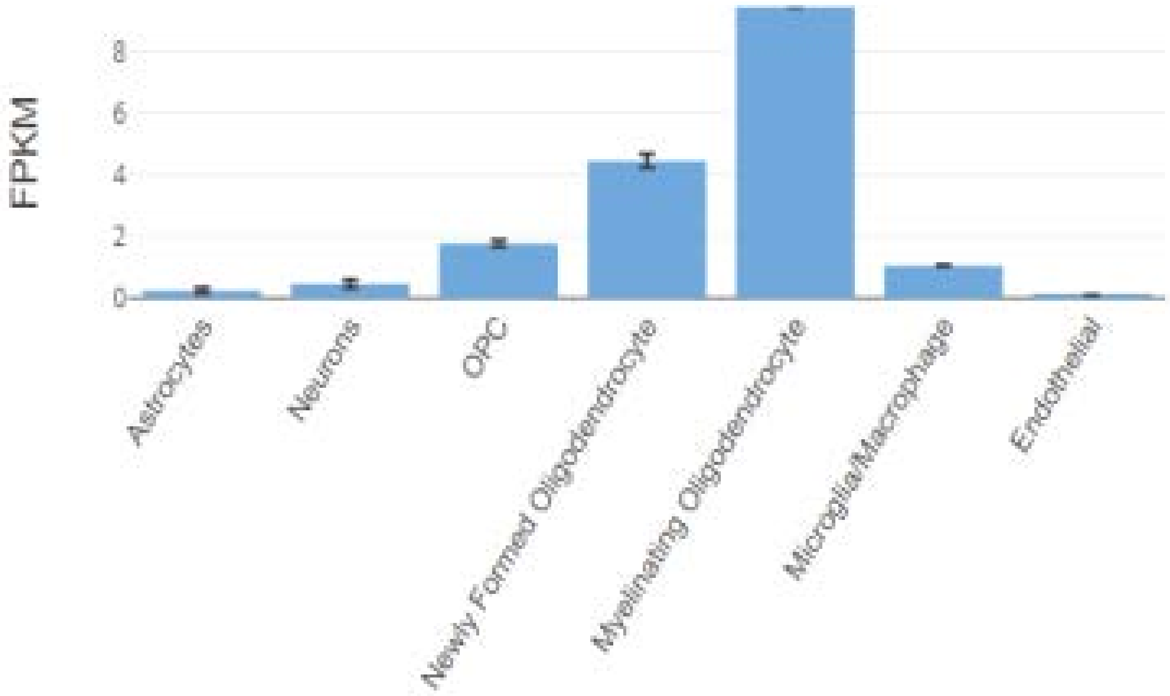

Gsn - Mus musculus

#13

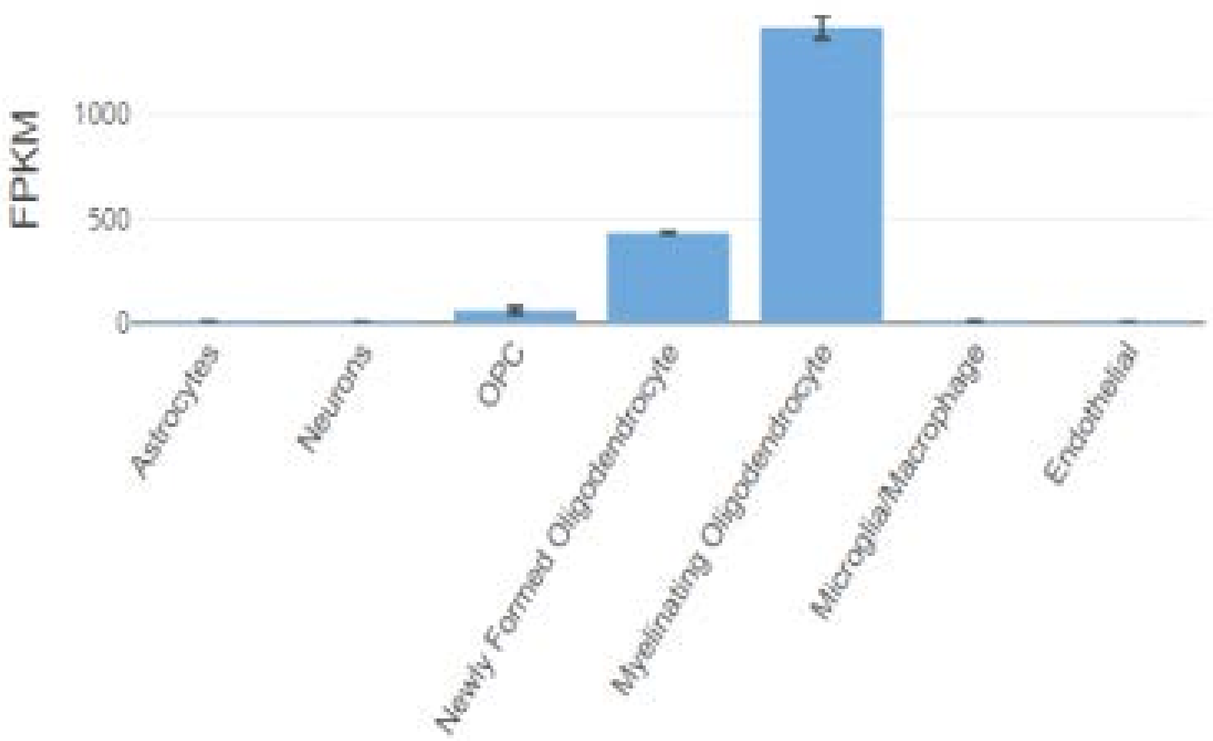

Gamt - Mus musculus

#14

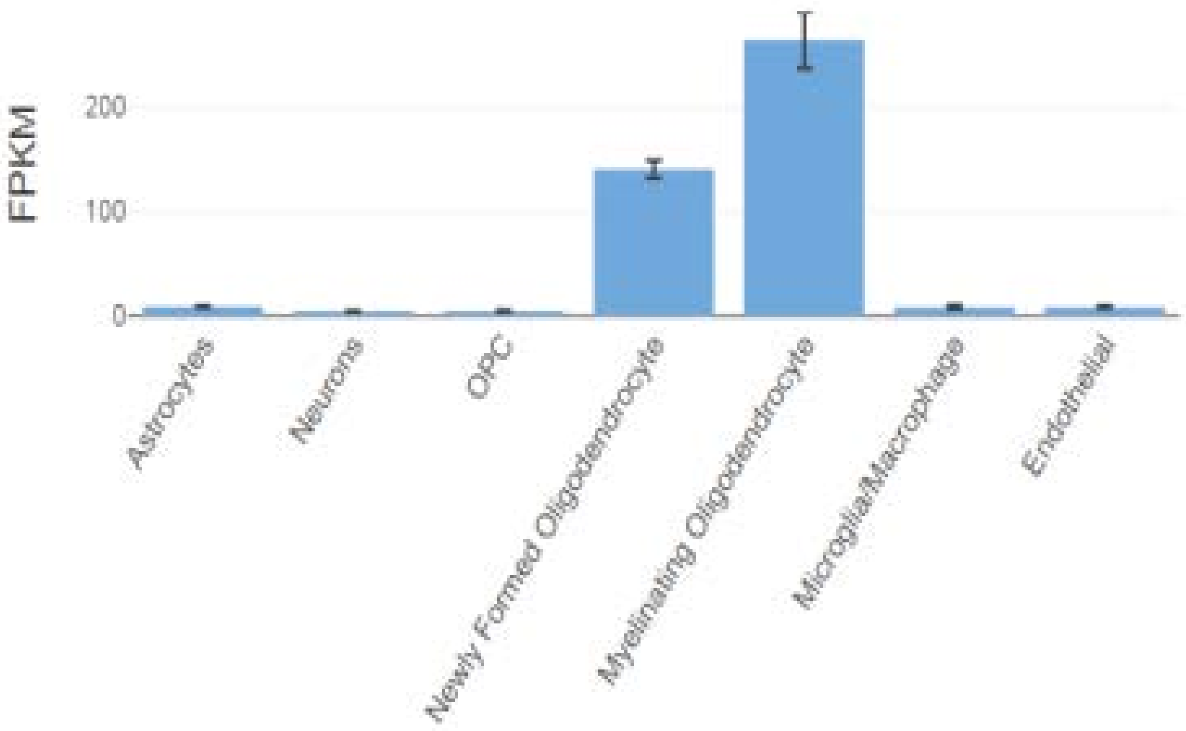

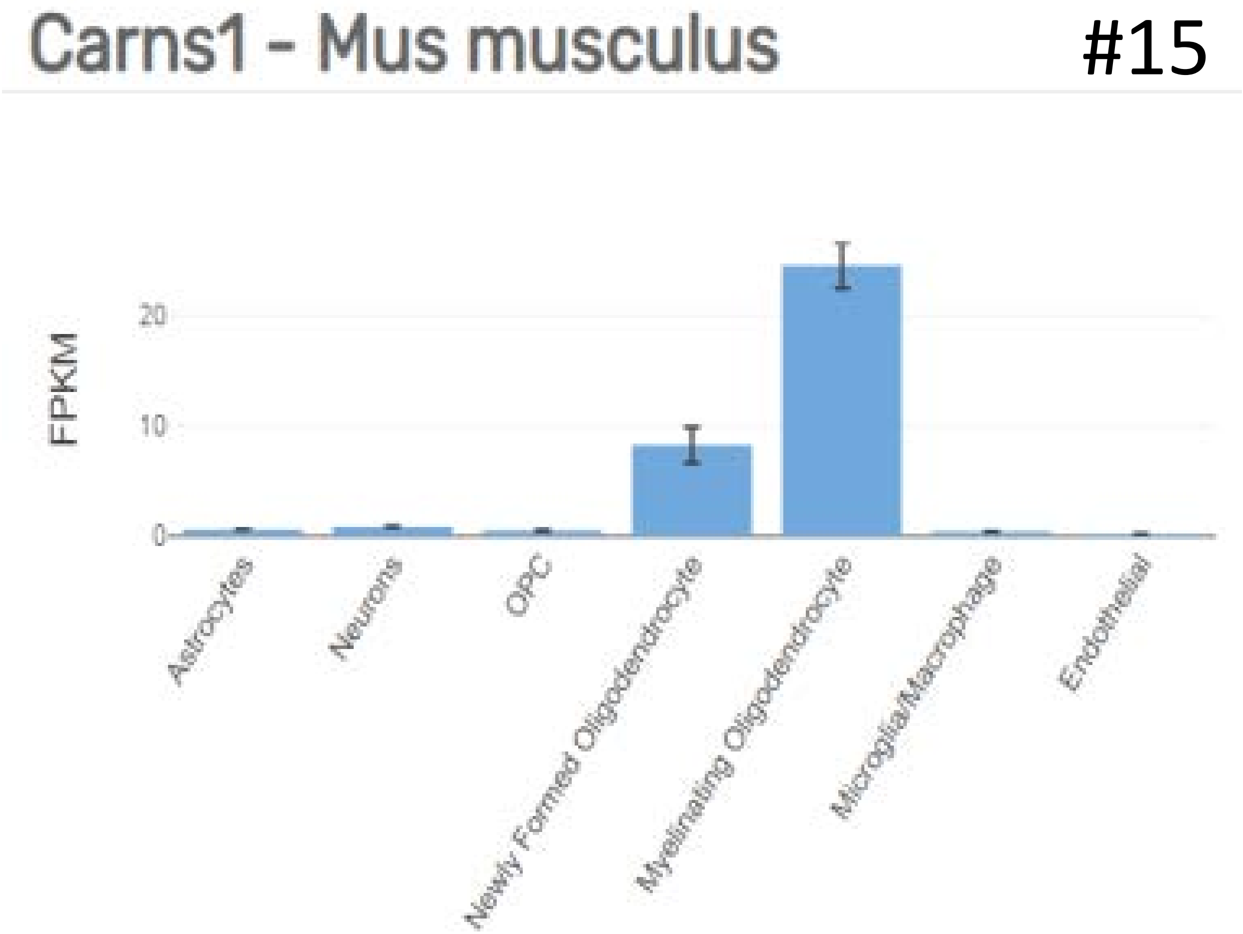
Carns1 - Mus musculus
#15
FPKM
20
10
0
Astrocytes
Neurons
OPC
Newly Formed Oligodendrocyte
Myelinating Oligodendrocyte
Microglia/Macrophage
Endothelial

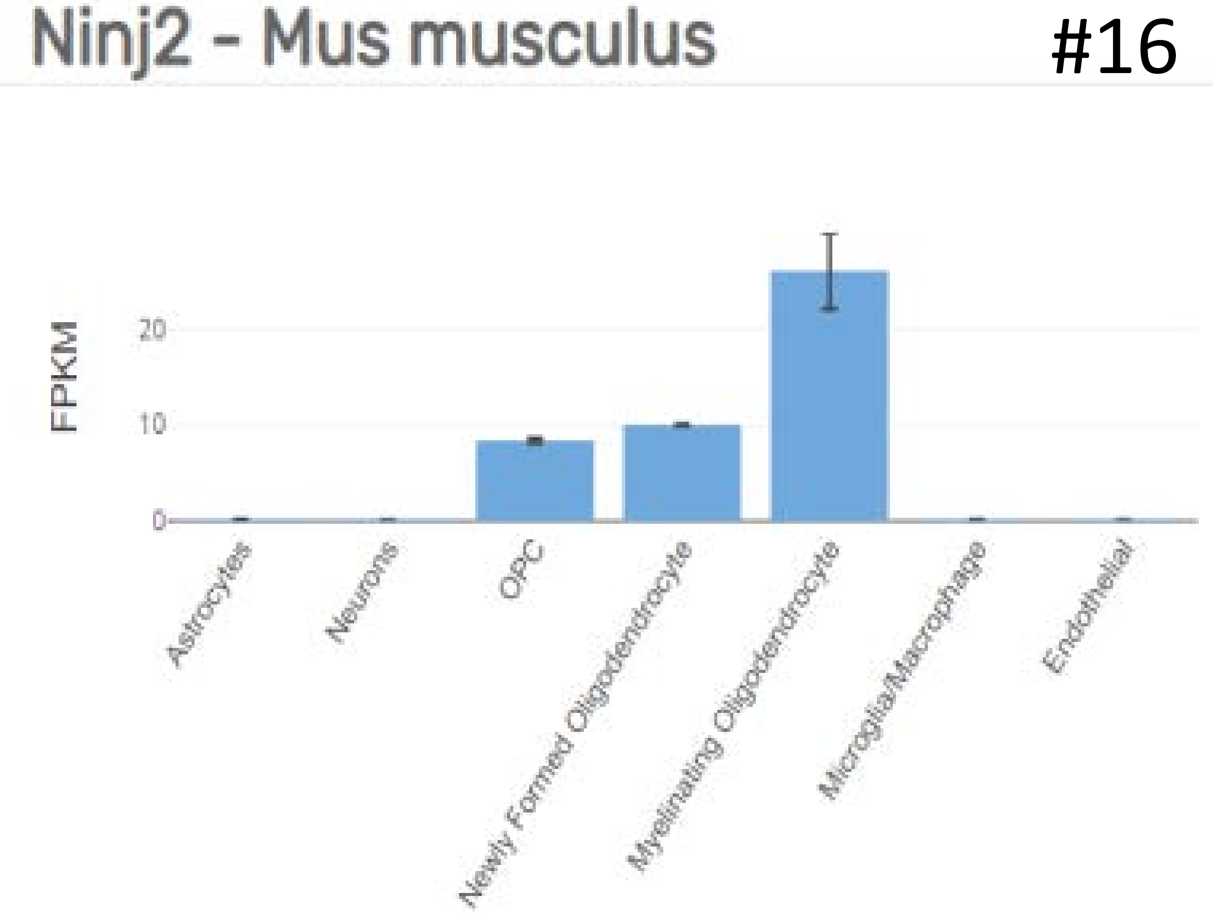
Ninj2 - Mus musculus
#16
FPKM
20
10
0
Astrocytes
Neurons
OPC
Newly Formed Oligodendrocyte
Myelinating Oligodendrocyte
Microglia/Macrophage
Endothelial

Thbs4 - Mus musculus #17

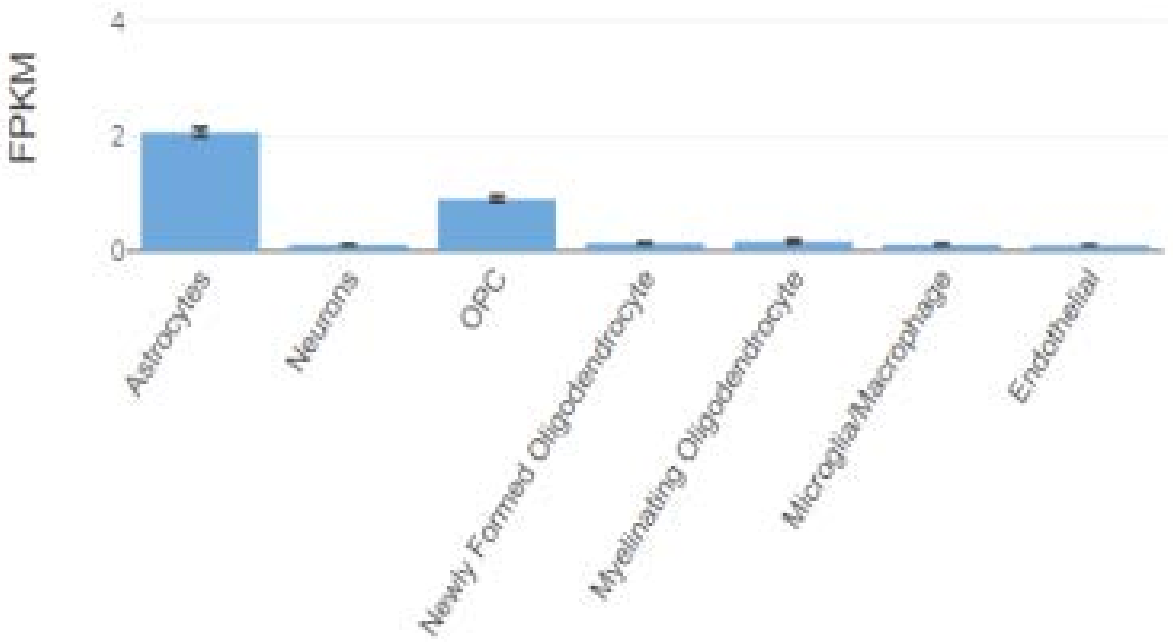

Ttyh2 - Mus musculus #18

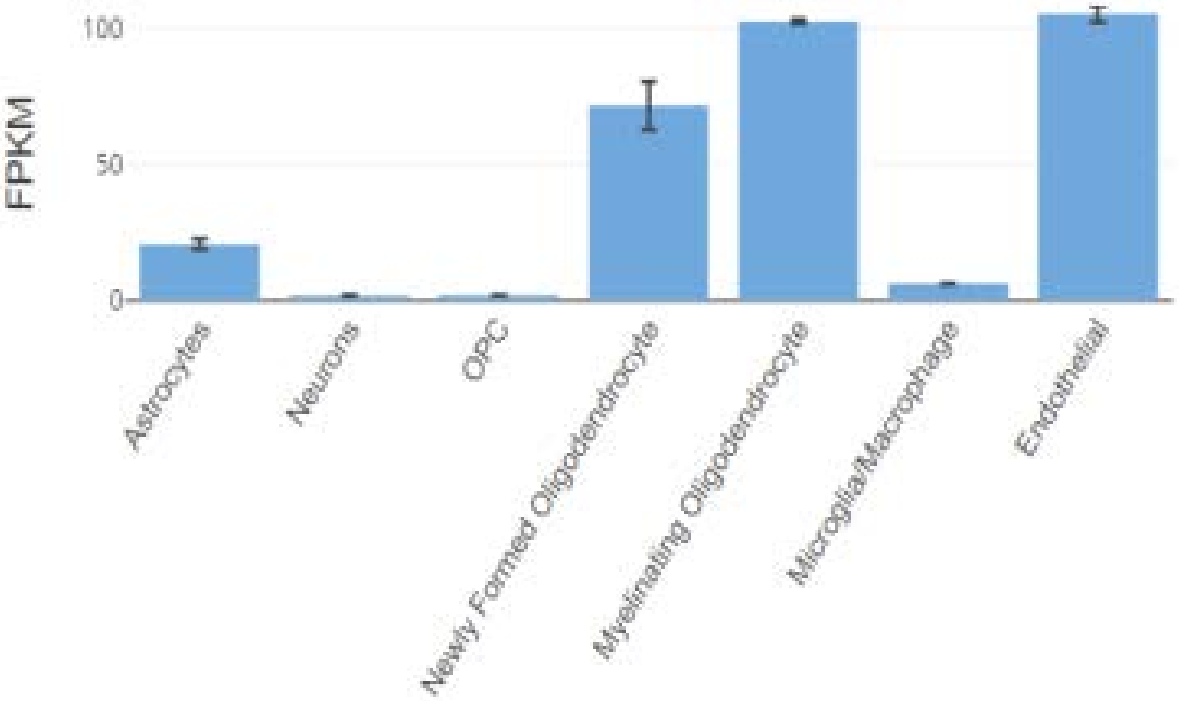

Cpm - Mus musculus

#19

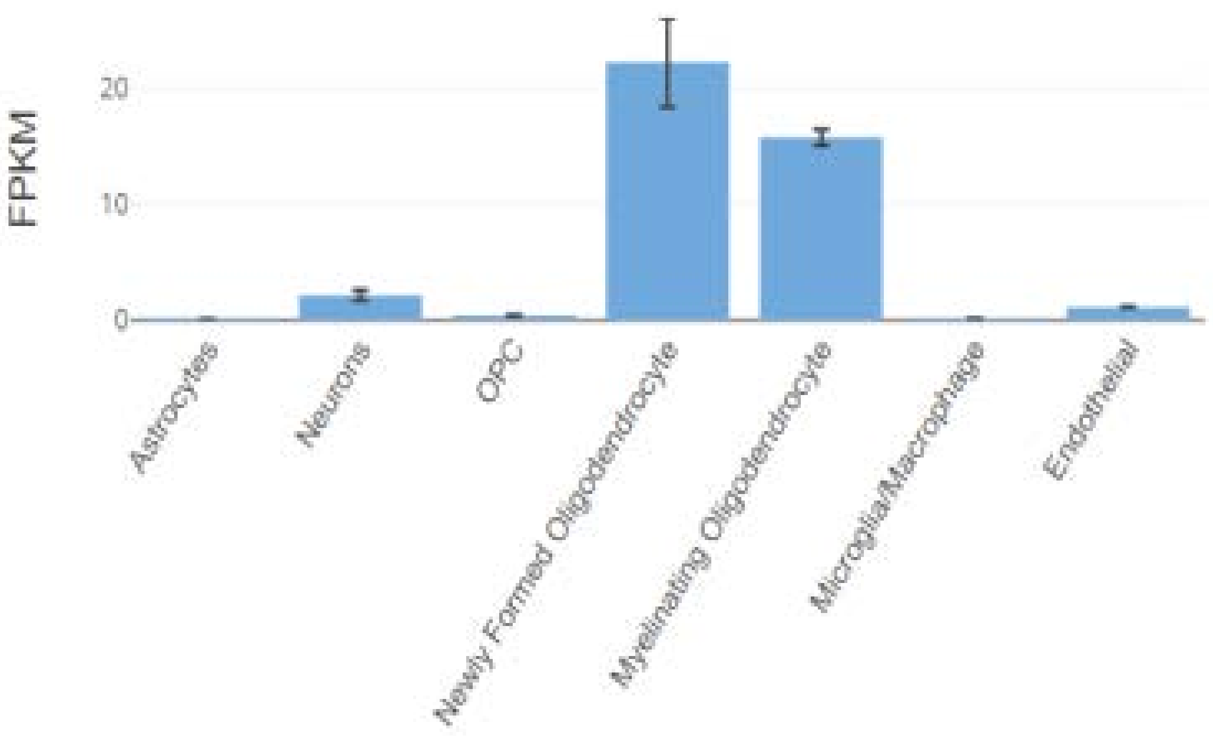

Gjc2 - Mus musculus

#20

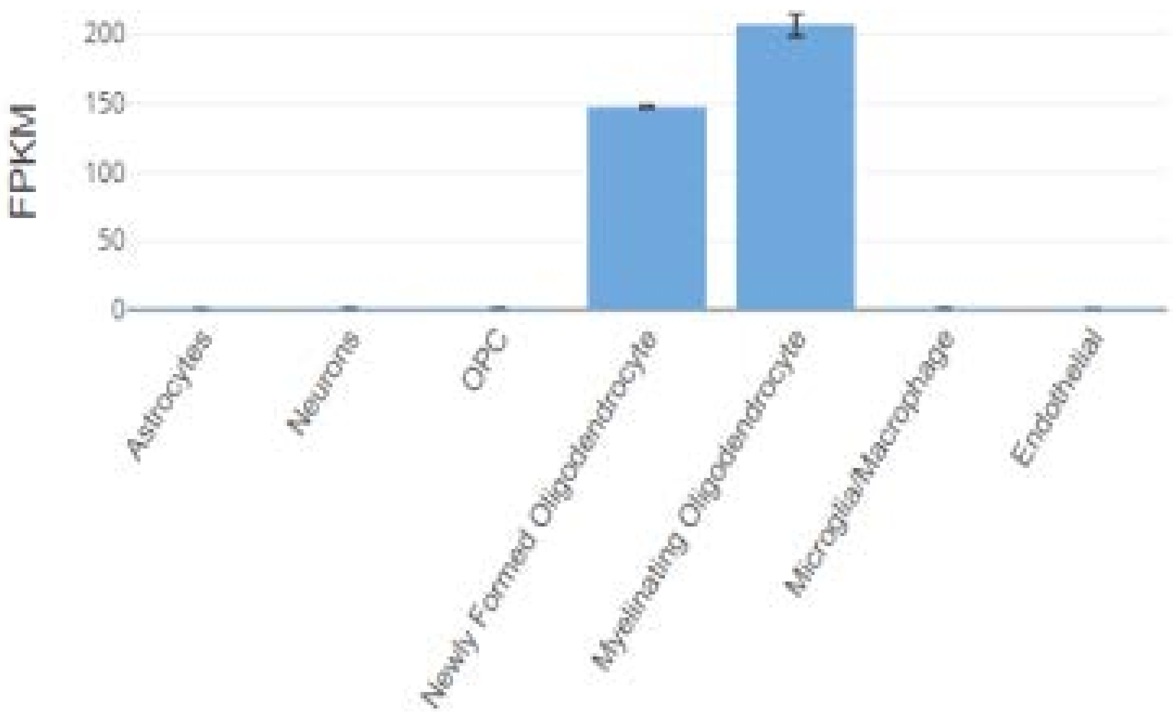

Tspan2 - Mus musculus

#21

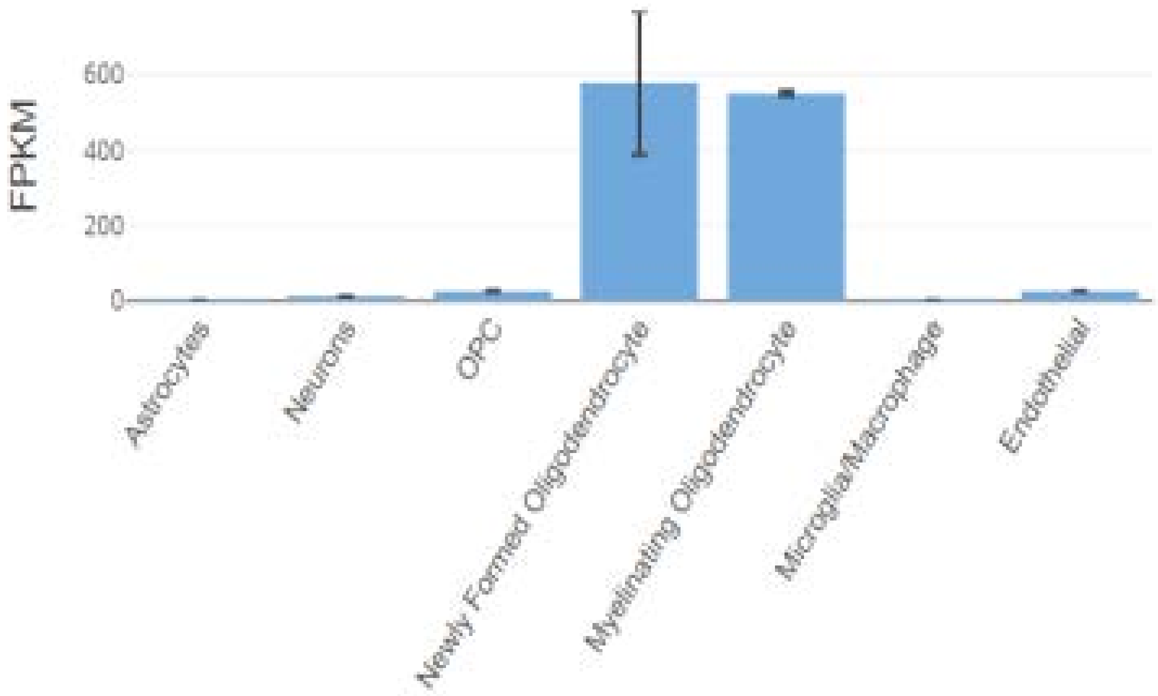

Nipal4 - Mus musculus

#22

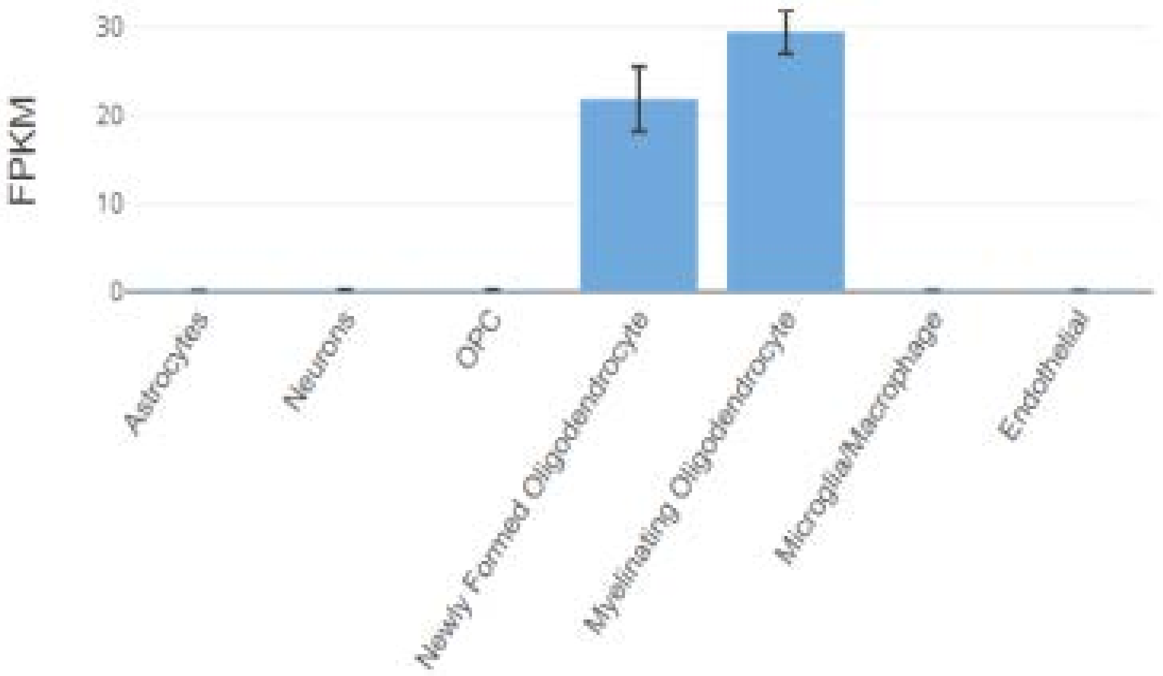

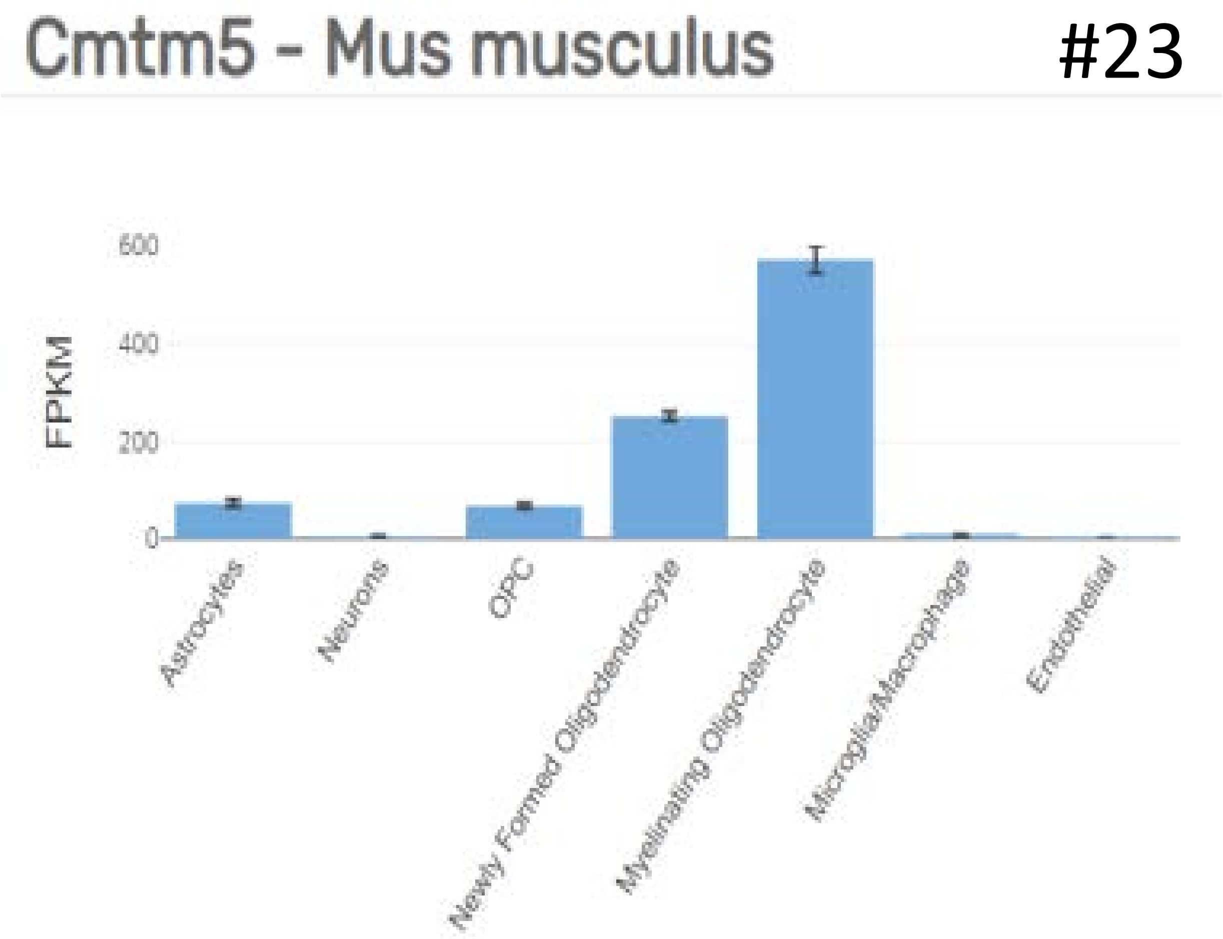
Cmtm5 - Mus musculus
#23
600
400
200
0
FPKM
Astrocytes
Neurons
OPC
Newly Formed Oligodendrocyte
Myelinating Oligodendrocyte
Microglia/Macrophage
Endothelial

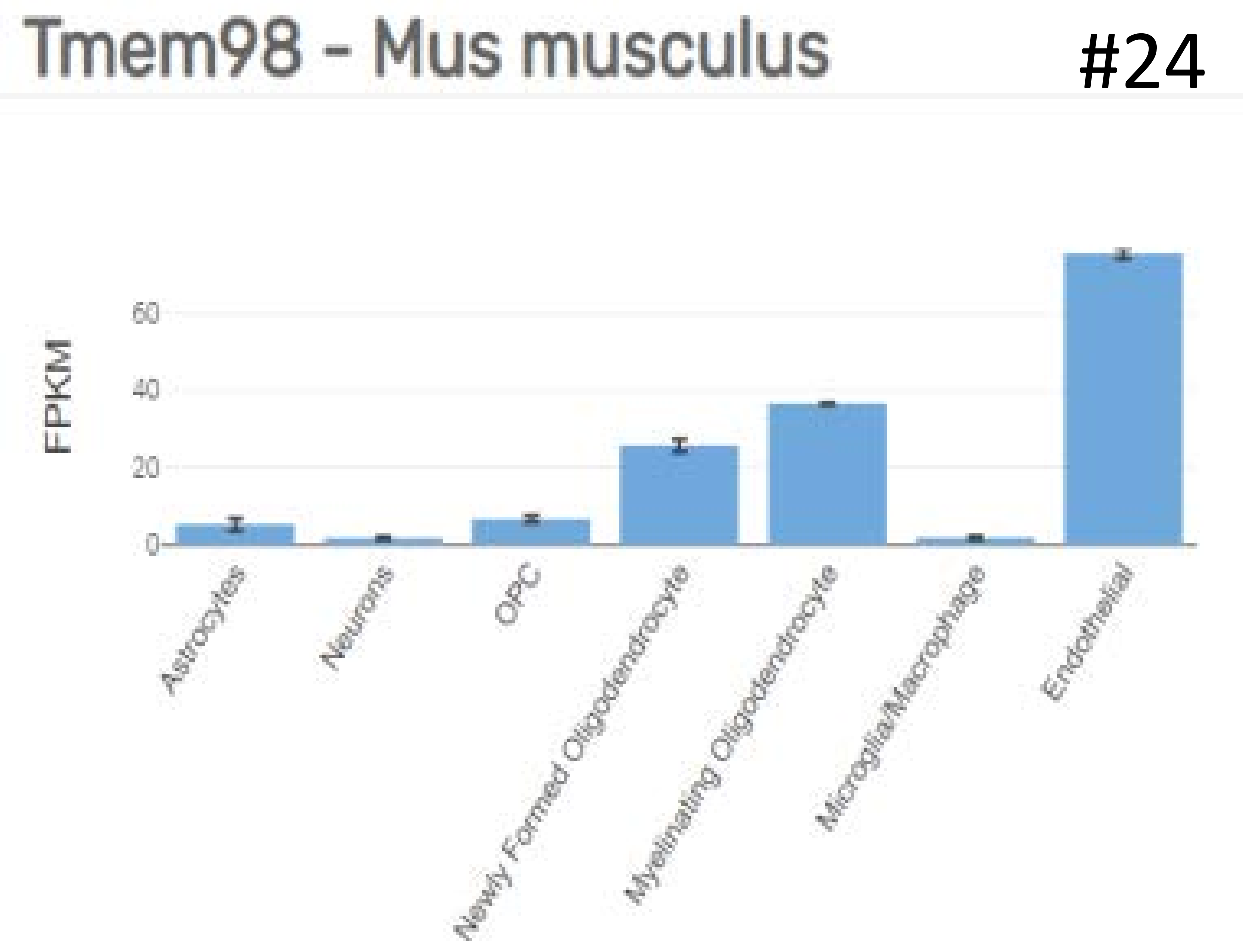
Tmem98 - Mus musculus
#24
60
40
20
0
FPKM
Astrocytes
Neurons
OPC
Newly Formed Oligodendrocyte
Myelinating Oligodendrocyte
Microglia/Macrophage
Endothelial

Plxnb3 - Mus musculus

#25

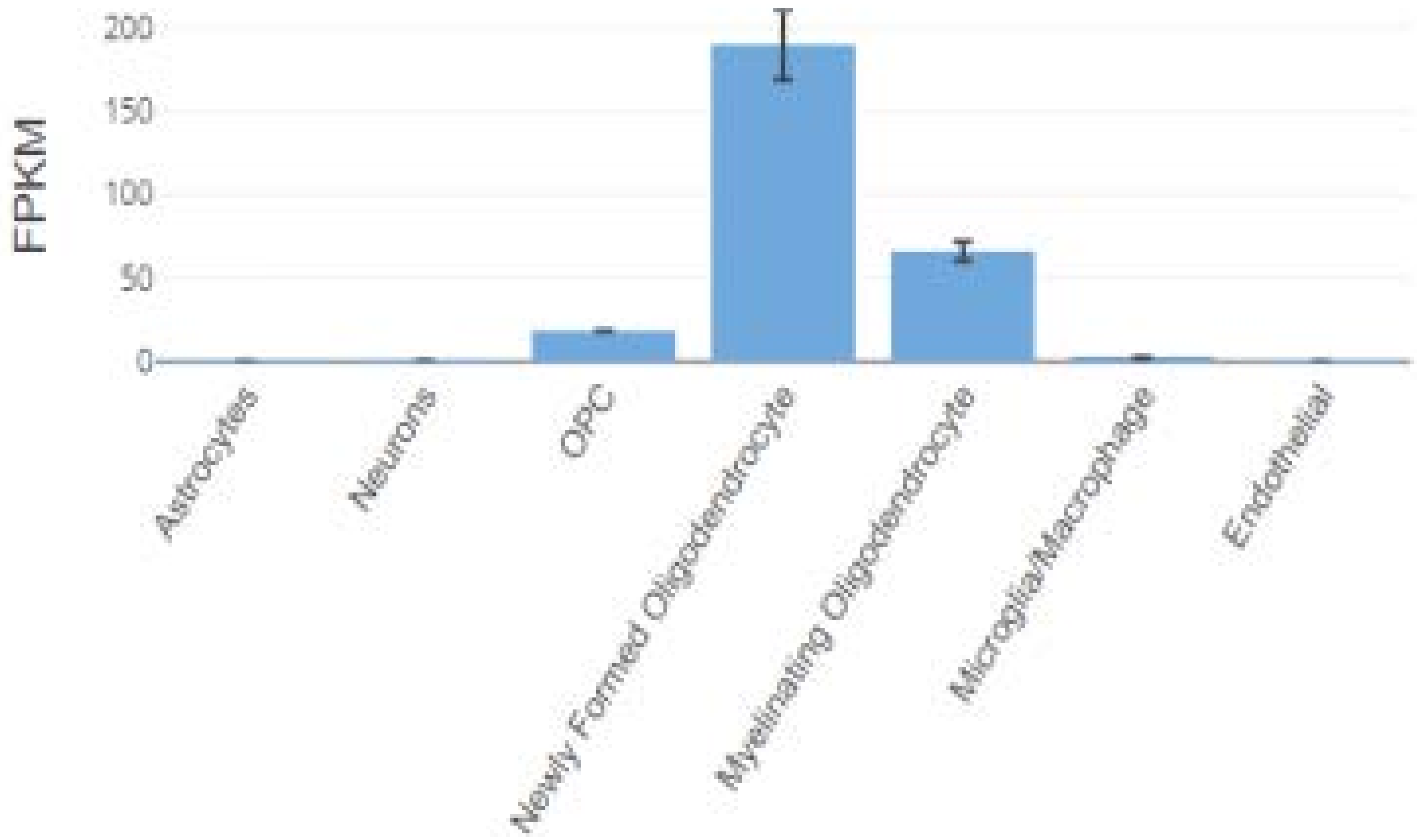

Supplement: Supplementary file 1 [file cells-09-00843-s001.pdf]
